# Membrane nanoclusters of FcγRI segregate from inhibitory SIRPα upon activation of human macrophages

**DOI:** 10.1083/jcb.201608094

**Published:** 2017-04-03

**Authors:** Filipa B. Lopes, Štefan Bálint, Salvatore Valvo, James H. Felce, Edith M. Hessel, Michael L. Dustin, Daniel M. Davis

**Affiliations:** 1 Manchester Collaborative Centre for Inflammation Research, University of Manchester, Manchester M13 9NT, England, UK; 2 Kennedy Institute of Rheumatology, University of Oxford, Oxford OX3 7FY, England, UK; 3 Refractory Respiratory Inflammation Discovery Performance Unit, GlaxoSmithKline, Hertfordshire SG1 2NY, England, UK

## Abstract

Signal integration between activating Fc receptors and inhibitory signal regulatory protein α (SIRPα) controls macrophage phagocytosis. Here, using dual-color direct stochastic optical reconstruction microscopy, we report that Fcγ receptor I (FcγRI), FcγRII, and SIRPα are not homogeneously distributed at macrophage surfaces but are organized in discrete nanoclusters, with a mean radius of 71 ± 11 nm, 60 ± 6 nm, and 48 ± 3 nm, respectively. Nanoclusters of FcγRI, but not FcγRII, are constitutively associated with nanoclusters of SIRPα, within 62 ± 5 nm, mediated by the actin cytoskeleton. Upon Fc receptor activation, Src-family kinase signaling leads to segregation of FcγRI and SIRPα nanoclusters to be 197 ± 3 nm apart. Co-ligation of SIRPα with CD47 abrogates nanocluster segregation. If the balance of signals favors activation, FcγRI nanoclusters reorganize into periodically spaced concentric rings. Thus, a nanometer- and micron-scale reorganization of activating and inhibitory receptors occurs at the surface of human macrophages concurrent with signal integration.

## Abstract (precis)

Lopes et al. use superresolution microscopy to visualize the nanoscale organization of activating and inhibitory receptors on human macrophages. Nanoclusters of inhibitory SIRPα and activating FcγRI associate in the cell’s resting state, but engagement of FcγRI induces their segregation.

## Introduction

Macrophages play a key role in immune defenses by phagocytosis of pathogens and other harmful foreign particles. Phagocytosis can be initiated by the binding of Fcγ receptors (FcγRs) to the constant Fc domain of IgG molecules on opsonized particles. This interaction induces lateral clustering of FcγRs (Jaumouillé and Grinstein, 2011) and phosphorylation by Src-family kinases (SFKs) of the immunoreceptor tyrosine-based activation motif (ITAM) present in the receptor’s cytosolic domain or on an associated common γ-chain (Ghazizadeh et al., 1994; Wang et al., 1994). Phosphorylation of the ITAM induces reorganization of the actin cytoskeleton, formation of a phagocytic synapse, and engulfment of the target particle (Flannagan et al., 2012).

FcγRs are presumed to exist as monomers and be evenly distributed on the cell surface (Jaumouillé et al., 2014). Upon interaction with their ligand, FcγRs are thought to diffuse through the surface membrane to accumulate around a particle by a zipper-like mechanism (Griffin et al., 1975). Serial binding of Fc receptors to ligands extends a pseudopod along the particle, and integrins aid its progression and closure of the phagocytic cup (Freeman et al., 2016). Triggering of this process is dependent on the balance of signals from specific activating and inhibitory receptors.

Signal regulatory protein α (SIRPα) negatively regulates macrophage phagocytosis (Fujioka et al., 1996; Veillette et al., 1998; Jiang et al., 1999; Oldenborg et al., 2001; Okazawa et al., 2005) by interacting with CD47, essentially a “marker of self” (Oldenborg et al., 2000). Ligation of the commonly expressed form of CD47 results in phosphorylation of the immunoreceptor tyrosine-based inhibition motif (ITIM) on the cytoplasmic tail of SIRPα, which leads to the recruitment of the tyrosine phosphatase SHP-1. This blocks phagocytosis, at least in part by preventing the accumulation of myosin-II A at the phagocytic synapse (Tsai and Discher, 2008).

Recently, superresolution microscopy has established that many receptors are clustered at the plasma membrane on a nanometer scale (Garcia-Parajo et al., 2014) and that the nanoscale organization of T cell, natural killer cell, or B cell surfaces changes when cells are activated (Lillemeier et al., 2010; Mattila et al., 2013; Pageon et al., 2013; Kläsener et al., 2014; Oszmiana et al., 2016). In macrophages, confocal fluorescence microscopy has established that both the inhibitory receptor SIRPα and the activating FcγRI accumulate at the phagocytic cup upon binding to their cognate ligands (Tsai and Discher, 2008; Yamauchi et al., 2012). Here, we used dual-color direct stochastic optical reconstruction microscopy (dSTORM; Heilemann et al., 2008) to visualize the spatial organization of the activating FcγRs and the inhibitory receptor SIRPα with a lateral resolution of 25 nm on human macrophages. This revealed an unexpected nanoscale and microscale rearrangement of macrophage cell surface receptors concurrent with signal integration.

## Results

### FcγRI and SIRPα are arranged in discrete nanoclusters at macrophage surfaces

**Figure 1.**
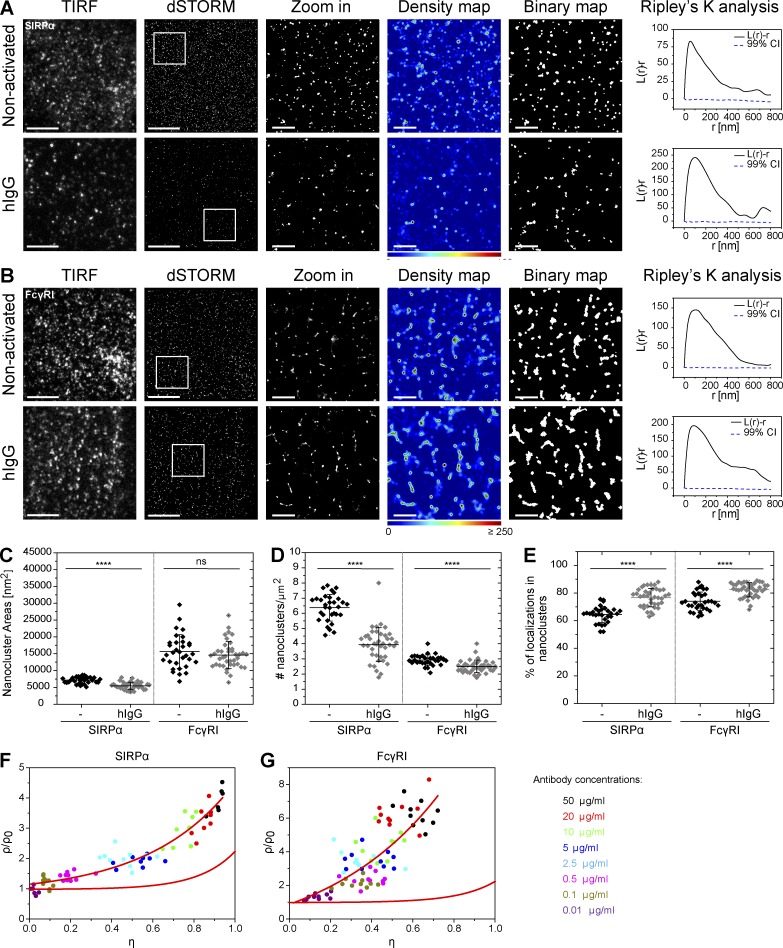
**SIRPα and FcγRI are arranged in discrete nanoclusters at macrophage surfaces.** (A and B) TIRF and dSTORM images of SIRPα (A) and FcγRI (B) at the surface of human macrophages seeded onto PLL- (nonactivated, top) or hIgG-coated slides (bottom) for 10 min and stained with fluorescently labeled specific antibodies. Bars, 5 µm. Regions delineated by white squares are zoomed-in and shown with corresponding density maps (pseudocolor scale), thresholded binary maps and Ripley’s K analysis of the molecules in the selected regions. Bars, 1 µm. L(r)-r represents the degree of clustering relative to simulated random distributions, indicated by the 99% confidence intervals (CIs); r is the radial scale. (C–E) Nanocluster areas (C), density (D), and percentage of localizations in nanoclusters (E) for SIRPα and FcγRI under nonactivating (black) or hIgG-activating (gray) conditions were calculated by subjecting dSTORM data to spatial point-pattern analysis and thresholding. Each symbol represents the median of several 5 × 5 µm regions from the same cell. Horizontal lines and error bars represent mean ± SD. Data are from a minimum of 30 cells from three independent donors. ns, not significant; ****, P < 0.0001; two-tailed *t* test assuming unequal variance. (F and G) Label-density variation analysis for SIRPα (F) and FcγRI (G) yields characteristic normalized ρ/η curves for clustered proteins. Cells were stained with anti–SIRPα-AF647 (F) or anti–FcγRI-AF488 (G) at different labeling concentrations and imaged by dSTORM. Each data point represents a single cell, color-coded by antibody concentration used for labeling. Red lines indicate reference curves for a random distribution of molecules.

To determine the organization of the inhibitory receptor SIRPα and the high-affinity Fc receptor FcγRI at a nanometer scale, we used the superresolution microscopy technique dSTORM. For this, human monocyte–derived macrophages (hereafter referred to as primary macrophages; phenotyped in Fig. S1 A) were plated onto poly-l-lysine (PLL)–coated slides (nonactivating condition) for 10 min and then fixed and stained with directly labeled anti–SIRPα or anti-FcγRI mAbs (Fig. 1). dSTORM images, along with Ripley’s K analysis (Ripley, 1977), of cells seeded under nonactivating conditions revealed that both receptors are not randomly distributed but instead organized in discrete and spatially separated nanoclusters at the cell surface (Fig. 1, A and B, top).

We next assessed the distribution of the receptors when cells were seeded onto glass slides coated with human IgG (hIgG), the ligand for FcγRs. This 2D model of the phagocytic synapse creates a flattened focal plane amenable for dSTORM. Cells were plated for 10 min on slides coated with 10 µg/ml hIgG and then fixed and stained as before. Upon activation of FcγRs, both FcγRI and SIRPα were predominantly organized in specific nanoclusters (Fig. 1, A and B, bottom).

To gain quantitative insight, we reconstituted the superresolution images as probability density maps of the molecules based on univariate Getis and Franklin’s local point pattern analysis (Getis and Franklin, 1987; Williamson et al., 2011). After thresholding, density maps were converted to binary maps in which regions containing dense localizations of the receptors appear white (referred to as nanoclusters; Fig. 1, A and B). Quantitative analysis revealed that SIRPα is constitutively arranged in nanoclusters with a mean area of 7,150 ± 980 nm^2^ (Fig. 1 C), corresponding to a radius of 48 ± 3 nm (assuming nanoclusters are circular), and a mean density of 6.4 ± 1 nanoclusters/µm^2^ (Fig. 1 D). These became smaller (5,440 ± 1,095 nm^2^, 42 ± 4 nm radius; Fig. 1 C) and less dense (3.9 ± 1 nanoclusters/µm^2^; Fig. 1 D) after activation through FcγRI. In contrast, the proportion of localizations in nanoclusters (i.e., the number of localizations in nanoclusters divided by the total number of localizations) increased slightly (mean of 64 ± 6% in nonactivating conditions to 77 ± 7% after activation; Fig. 1 E). FcγRI constitutively forms larger (mean area of 15,650 ± 5,030 nm^2^, 71 ± 11 nm radius) and less dense (mean of 2.9 ± 0.3 nanoclusters/µm^2^) nanoclusters than SIRPα, containing a higher proportion of localizations (mean of 74 ± 7%). The size (14,610 ± 4,030 nm^2^, 68 ± 9 nm radius) of FcγRI nanoclusters remained the same, whereas their density (2.5 ± 0.4 nanoclusters/µm^2^) decreased upon stimulation with hIgG (Fig. 1, C and D), and the proportion of localizations in nanoclusters increased (82 ± 5%; Fig. 1 E). Flow cytometry showed a decrease of 8% and 19% in geometric mean fluorescence intensity for SIRPα and FcγRI, respectively, after activation of cells with surface-immobilized hIgG for 10 min (Fig. S1, B and C), suggesting a small fraction of both receptors are internalized from the interface.

An alternative method of analysis to discriminate clustered from randomly distributed molecules is based on deliberate variation of labeling density combined with cluster analysis and is insensitive to artifacts generated by overcounting of blinking fluorophores (Baumgart et al., 2016). To apply this here, SIRPα and FcγRI were labeled with a range of concentrations (0.01–50 µg/ml) of directly labeled antibodies and imaged by dSTORM. For each image, the relative area covered by the cluster masks (η), obtained from thresholded binary maps, and the mean density of localizations within the clusters (ρ) were calculated. As Baumgart et al. (2016) discussed in detail, clustered and random distributions can be discriminated by plotting the normalized density ρ/ρ_0_ (where ρ_0_ is the intersection of the density curves with the y axis) against η. For randomly distributed molecules, a horizontal line is observed, whereas for clustered receptors, there is an increase in ρ/ρ_0_. By this analysis, SIRPα and FcγRI are clustered at the surface of primary human macrophages, as ρ/ρ_0_ increases when the labeling concentration increases (Fig. 1, F and G). Altogether, these data show that the inhibitory receptor SIRPα and the high-affinity Fc receptor FcγRI are organized in membrane nanoclusters at the surface of primary human macrophages before and after activation of FcγRs.

### Nanoclusters of SIRPα and FcγRI are constitutively associated but segregate upon stimulation with hIgG

**Figure 2.**
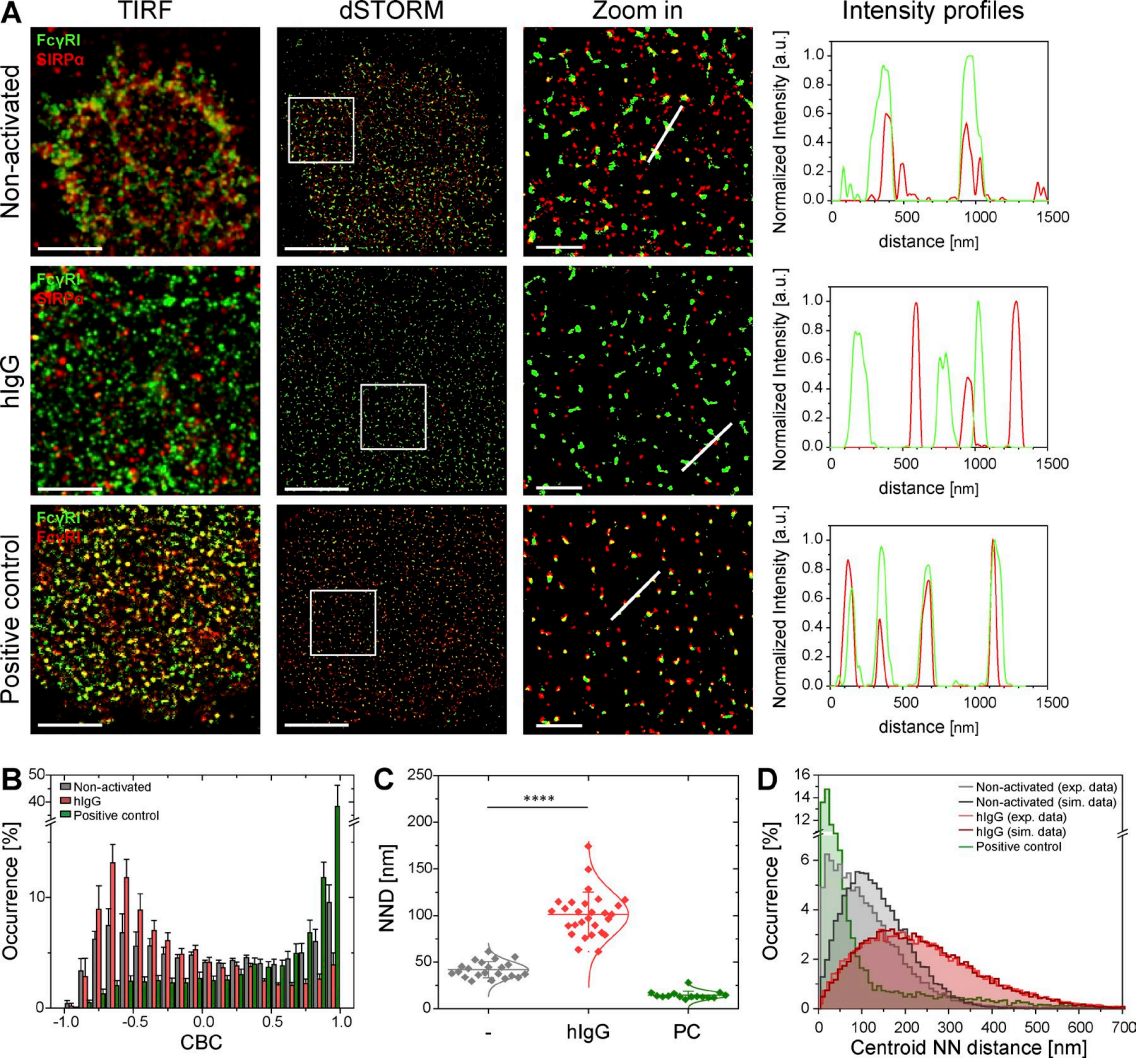
**SIRPα and FcγRI nanoclusters are constitutively associated in nonactivated human macrophages but segregate upon activation with hIgG.** (A) TIRF and dSTORM images showing FcγRI (green) and SIRPα (red) at the surface of human macrophages incubated for 10 min on slides coated with PLL (nonactivated, top) or hIgG (middle) and stained with anti–FcγRI-AF488 and anti–SIRPα-AF647 mAbs. Bars, 5 µm. Regions outlined by the white squares (middle column) are shown enlarged (right columns) with relative fluorescence intensity profiles along the white lines. Bars, 1 µm. As a positive control, macrophages seeded onto PLL-coated slides were stained with anti–FcγRI-AF488 mAb followed by anti–mouse IgG1-AF647 secondary antibody (bottom). (B) CBC histograms of the single-molecule distributions of the colocalization parameter for SIRPα and FcγRI in cells seeded onto PLL- (gray) or hIgG-coated (red) slides for 10 min or for positive control data (green). Data are from a minimum of 30 cells from three independent donors. Bars represent mean ± SD. (C) Nearest-neighbor (NN) analysis from data shown in (B). Each symbol represents the median NN of all paired single-molecule localizations from one cell. Horizontal lines and error bars represent mean ± SD. ****, P < 0.0001; two-tailed *t* test assuming unequal variance. (D) Histogram distributions of the NND between the centroids of nanoclusters from one channel and the centroid of their nearest neighbor from the second channel (≥ 20,000 clusters from a minimum of 10 cells per condition) from cells seeded onto PLL- (light gray), hIgG-coated (light red) slides, or positive control data (green). Corresponding simulated data are also shown, in which the centroid positions of SIRPα nanoclusters in both nonactivating (dark gray) and hIgG-activating conditions (dark red) were randomized within the cell area.

We next set out to use dual-color dSTORM to investigate the relationship between SIRPα and FcγRI nanoclusters. To validate our imaging and analysis, a positive control for colocalization was included in which the same protein, FcγRI, was stained with a primary anti–FcγRI mAb conjugated with Alexa Fluor 488 (AF488) followed by isotype-specific secondary antibody conjugated with an alternative fluorophore, AF647 (Fig. 2 A, bottom).

To examine the organization of SIRPα and FcγRI nanoclusters, cells were plated under nonactivating conditions for 10 min before being fixed and stained with anti–FcγRI-AF488 and anti–SIRPα-AF647 mAbs. Dual-color dSTORM revealed that SIRPα nanoclusters are constitutively associated with FcγRI nanoclusters at the surface of primary human macrophages (Fig. 2 A, top). The degree of colocalization between the two receptors was addressed by subjecting SIRPα and FcγRI localization lists to coordinate-based colocalization (CBC) analysis (Malkusch et al., 2012), which assigns a correlation coefficient to each single localization of each protein within a certain radial distance, ranging from −1 (perfectly segregated) to 0 (uncorrelated distributions) to +1 (perfectly colocalized).

CBC analysis showed that, in nonactivated cells, a large proportion of SIRPα is associated with FcγRI within a search radius of 50 nm, as demonstrated by the histogram distribution of the colocalization parameter being distributed toward +1 (50% of localizations are between 0 and 1; Fig. 2 B). The search radius of 50 nm was chosen based on the mean radius of SIRPα nanoclusters in nonactivating conditions. Corroborating this, the mean nearest-neighbor distance (NND) of paired single-molecule localizations was 42 ± 9 nm (Fig. 2 C). A proportion of SIRPα (36 ± 6%) and FcγRI (26 ± 7%) molecules are not localized within nanoclusters (Fig. 1 E), which could lower the degree of colocalization in CBC analysis. To test directly whether or not nanoclusters associate, we measured the NND between the centroids of nanoclusters of the two receptors. The centroid NND between SIRPα and FcγRI nanoclusters has a mode of 62 ± 5 nm (Fig. 2 D).

To test whether or not such close association between SIRPα and FcγRI nanoclusters occurs by chance, simply as an outcome of the density of nanoclusters at the cell surface, we next analyzed simulated images in which the centroid positions of SIRPα nanoclusters were randomized within the cell area. The mode of the centroid NND was 122 ± 2 nm for these simulated images, compared with 62 ± 5 nm in experimental data (Fig. 2 D). This confirms a specific association between SIRPα and FcγRI nanoclusters in nonactivated macrophages.

Upon activation of FcγRI, the two receptors segregated from each other (Fig. 2 A, middle row), as indicated by an increase in the proportion of SIRPα molecules with negative correlation coefficients (toward −1; Fig. 2 B) and a significant increase in the NND between individual localizations of both receptors (to a mean of 101 ± 24 nm; Fig. 2 C). Moreover, the centroid NND between SIRPα and FcγRI nanoclusters increased (Fig. 2 D). Indeed, the mode for the centroid NND between SIRPα and FcγRI nanoclusters, upon FcγRI ligation, was 197 ± 3 nm and 207 ± 3 nm for experimental and simulated data, in which the positions of SIRPα nanoclusters were randomized, respectively. In contrast, CBC analysis of the positive control–stained cells showed a very high degree of colocalization (Fig. 2 B) with a mean NND of paired single-molecule localizations of 15 ± 4 nm (Fig. 2 C) and a mode for the centroid NN distances between nanoclusters of 28 ± 1 nm (Fig. 2 D). After 30 min of stimulation, there was no significant difference in any parameter describing the organization of SIRPα or FcγRI nanoclusters (Fig. S1, B and C; and Fig. S2), showing that segregation of these nanoclusters persists. Thus, upon ligation of FcγRI, SIRPα, and FcγRI nanoclusters are no longer associated and become distributed independently.

### Nanoclusters of SIRPα and FcγRII are constitutively segregated

**Figure 3.**
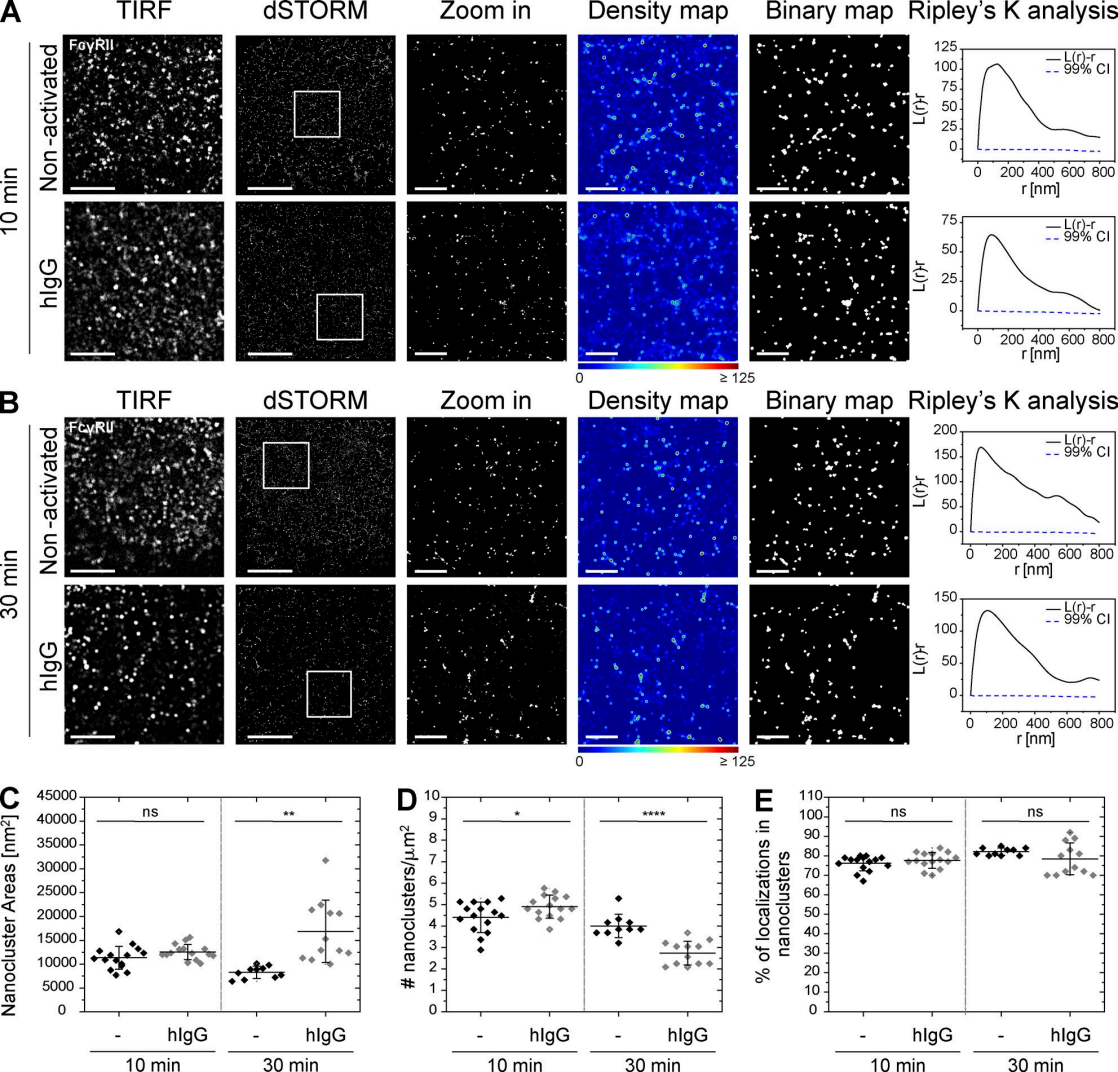
**The low-affinity Fc receptor FcγRII is arranged in discrete nanoclusters at macrophage surfaces.** (A and B) TIRF and dSTORM images of FcγRII at the surface of human macrophages seeded onto PLL- (nonactivated) or hIgG-coated slides for 10 min (A) or 30 min (B) and stained with a fluorescently labeled specific antibody. Bars, 5 µm. Regions delineated by the white squares are zoomed-in and shown with corresponding density maps, binary maps, and Ripley’s K analysis. Bars, 1 µm. (C–E) Nanocluster areas (C), density (D), and percentage of localizations in nanoclusters (E) for FcγRII under nonactivated (black) or hIgG-activated (gray) conditions at 10 or 30 min were calculated as in Fig. 1. Horizontal lines and error bars represent mean ± SD. Data are from a minimum of 15 cells from three independent donors. ns, not significant; *, P < 0.05; **, P < 0.01; ****, P < 0.0001; two-tailed *t* test assuming unequal variance.

To test if the association between SIRPα and FcγRI nanoclusters is specific, we next studied the relationship between SIRPα and the low-affinity activating Fc receptor FcγRII (CD32). Similar to SIRPα and FcγRI, FcγRII also assembles in discrete nanoclusters at the surface of primary human macrophages, both constitutively and upon activation with hIgG (Fig. 3, A and B). Quantitative analysis showed that, in nonactivated cells, at 10 min, FcγRII nanoclusters have a mean area of 11,400 ± 2,400 nm^2^ (corresponding to a mean radius of 60 ± 6 nm), a mean density of 4.4 ± 1 nanoclusters/µm^2^, and a mean percentage of localizations in nanoclusters of 76 ± 4% (Fig. 3, C–E). These values remained largely unchanged after activation with hIgG for 10 min (Fig. 3, C–E). However, after 30 min of activation, FcγRII nanoclusters became larger (mean area of 16,900 ± 6,540 nm^2^, mean radius of 73 ± 13 nm; Fig. 3 C) and less dense (mean density of 2.7 ± 1 nanoclusters/µm^2^; Fig. 3 D). Flow cytometry showed no significant change in FcγRII expression levels after activation (Fig. S1 D).

**Figure 4.**
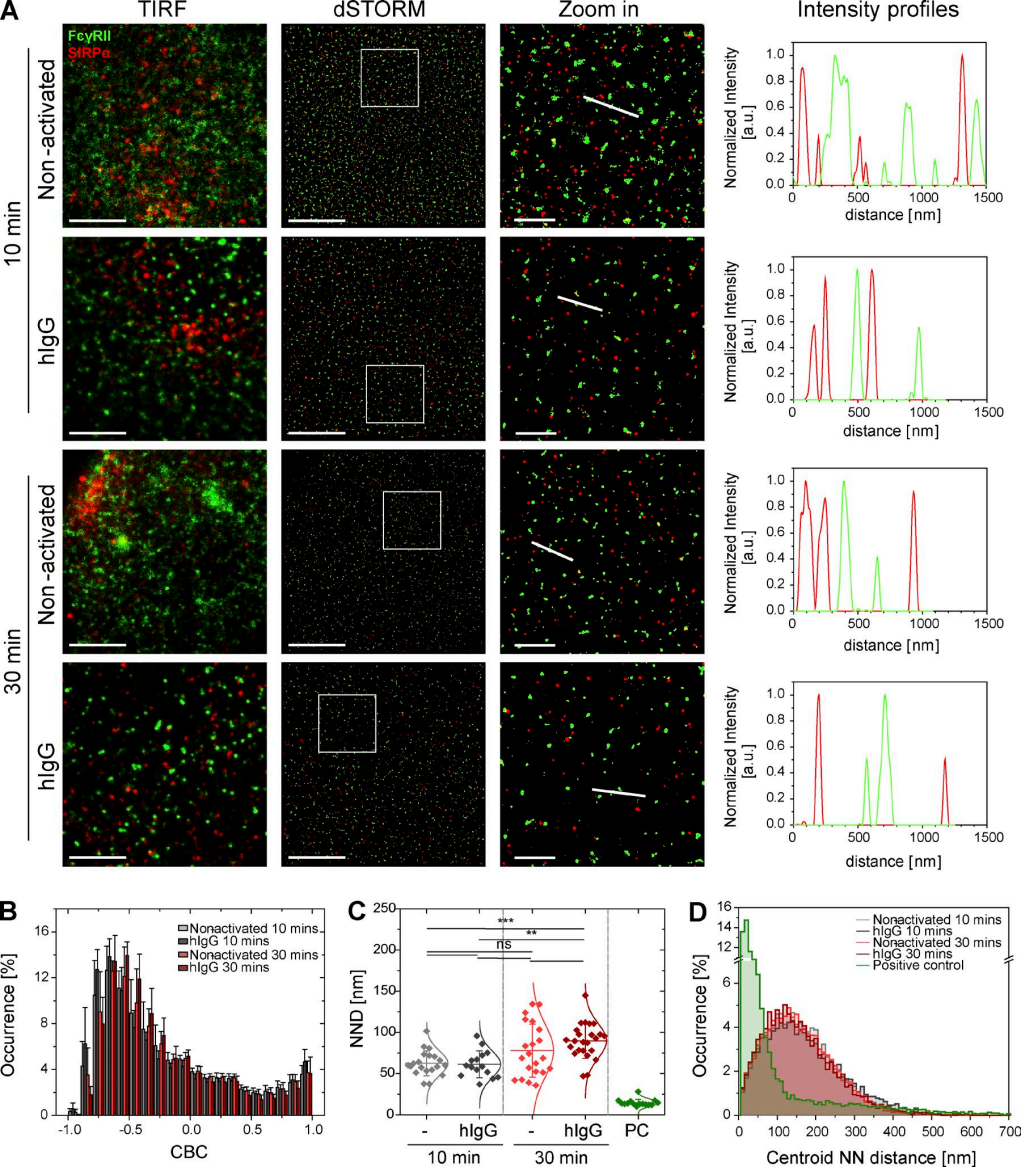
**SIRPα and the low-affinity Fc receptor, FcγRII, are segregated on a nanometer scale.** (A) TIRF and dSTORM images showing FcγRII (green) and SIRPα (red) at the surface of human macrophages incubated for 10 or 30 min on slides coated with PLL (nonactivated) or hIgG and stained with anti–FcγRII-AF488 and anti–SIRPα-AF647 mAbs. Bars, 5 µm. In each condition, regions outlined by the white squares (middle column) are shown enlarged (right column) with relative fluorescence intensity profiles along the white lines. Bars, 1 µm. (B) CBC histograms of the single-molecule distributions of the colocalization parameter for SIRPα and FcγRII in cells seeded onto PLL- or hIgG-coated slides for 10 (light gray and dark gray, respectively) or 30 min (light red and dark red, respectively) or for positive control data (green). The positive control data in this figure is the same as in Fig. 2. Data are from a minimum of 30 cells from three independent donors. Bars represent mean ± SD. (C) NND analysis from data shown in B. Each symbol represents the median NND of all paired single-molecule localizations from one cell. Horizontal lines and error bars represent mean ± SD. ns, not significant; **, P < 0.01; ***, P < 0.001; one-way analysis of variance (ANOVA) with Tukey’s post-hoc test. (D) Histogram distributions of the NND between the centroids of nanoclusters from one channel and the centroid of their nearest neighbor from the second channel (≥20,000 clusters from a minimum of 10 cells per condition). a.u. arbitrary units; NN, nearest neighbor; PC, positive control.

In contrast to FcγRI, FcγRII is not associated with SIRPα in nonactivated cells or upon activation (Fig. 4 A). This was confirmed by CBC analysis (distribution of the CBC histograms toward negative correlation coefficients and mean NND of paired single-localizations all >50 nm; Fig. 4, B and C) and by the centroid NND (with a mode of ∼140 nm in all conditions; Fig. 4 D). Altogether, these observations indicate that under nonactivating conditions, SIRPα is associated at the nanometer scale with the high-affinity Fc receptor FcγRI, but not with the low-affinity Fc receptor FcγRII, and upon activation, SIRPα and FcγRI nanoclusters segregate.

### FcγRI and FcγRII reorganize into concentric rings upon activation

**Figure 5.**
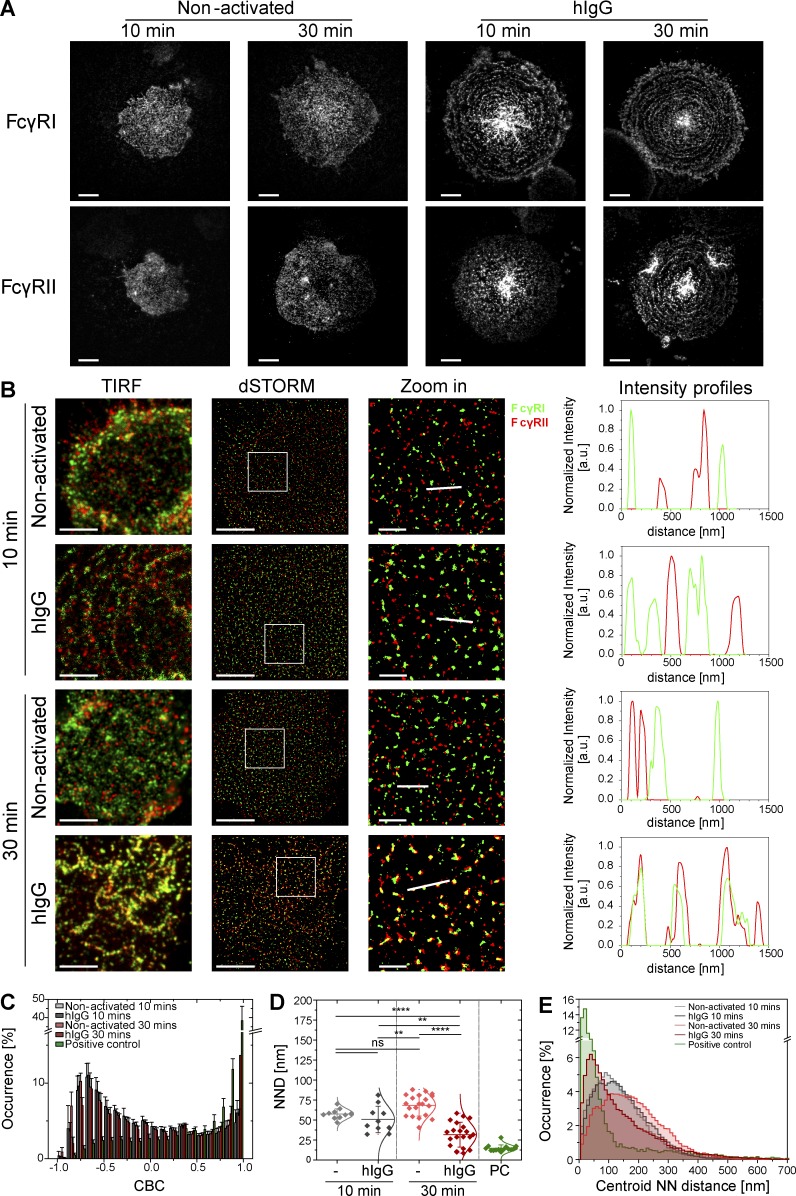
**FcγRs reorganize into concentric rings upon activation.** (A) TIRF images of FcγRI (top) and FcγRII (bottom) at the surface of human macrophages incubated for 10 or 30 min on slides coated with PLL (nonactivated) or hIgG and stained with fluorescently labeled specific antibodies. Bars, 10 µm. (B) TIRF and dSTORM images of FcγRI (green) and FcγRII (red) at the surface of macrophages incubated for 10 or 30 min on slides coated with PLL or hIgG and stained with anti–FcγRI-AF488 and anti–FcγRII-AF647 mAbs. Bars, 5 µm. Regions outlined by the white squares (middle column) are shown enlarged (right column) with relative fluorescence intensity profiles along the white lines. Bars, 1 µm. (C) CBC histograms of the single-molecule distributions of the colocalization parameter for FcγRI and FcγRII in cells seeded onto PLL- or hIgG-coated slides for 10 (light gray and dark gray, respectively) or 30 min (light red and dark red, respectively) or for positive control data (green). The positive control data in this figure are the same as in Fig. 2. Data are from a minimum of 10 cells from three independent donors. Bars represent mean ± SD. (D) NND analysis from data shown in C. Each symbol represents the median NND of all paired single-molecule localizations from one cell. Horizontal lines and error bars represent mean ± SD. ns, not significant; **, P < 0.01; ****, P < 0.0001; one-way ANOVA with Tukey’s post-hoc test. (E) Histogram distributions of the NND between the centroids of nanoclusters from one channel and the centroid of their nearest neighbor from the second channel (≥20,000 clusters from a minimum of 10 cells per condition). a.u., arbitrary units; NN, nearest neighbor; PC, positive control.

When plated onto IgG-coated slides, macrophages spread to a uniform radial morphology with multiple pseudopod extensions (Heiple et al., 1990; Cannon and Swanson, 1992). Here, total internal reflection fluorescence (TIRF) microscopy of primary human macrophages was used to examine the location of FcγRI and FcγRII within the morphology of activated cells (Fig. 5 A). Unexpectedly, both types of FcγR were found to reorganize into concentric rings at the surface of macrophages stimulated by slides coated with hIgG. Redistribution of the high-affinity Fc receptor, FcγRI, into concentric rings was detected as early as after 10 min of incubation, whereas that of the low-affinity Fc receptor, FcγRII, was visible after 30 min (Fig. 5 A). In contrast, when plated under nonactivating conditions, macrophages spread asymmetrically and FcγRs did not reorganize into concentric rings at any time of incubation (Fig. 5 A).

Live-cell TIRF microscopy was used to visualize formation of the ring structures. Cells were labeled in suspension with a directly conjugated nonblocking anti–FcγRI mAb and imaged as they landed onto nonactivating PLL-coated (Video 1) or activating hIgG-coated slides (Video 2). In nonactivating conditions, cell shape was irregular, and FcγRI nanoclusters remained homogeneously distributed at the cell surface. On slides coated with hIgG, cells spread rapidly and uniformly, and the formation of the rings was concurrent with cell spreading. Rings of FcγRI periodically assembled at the leading edge of the cell until spreading stopped.

Formation of the rings could result solely from the redistribution of the receptors already present at the synapse or, in addition, from the recruitment of proteins from other parts of the cell to the phagocytic synapse. To investigate this, we acquired confocal images of cells allowed to spread under nonactivating (Fig. S3 A) or hIgG-activating (Fig. S3 B) conditions for 10 min before fixation and staining for FcγRI. 3D confocal image reconstruction indicated that FcγRI is recruited to the activating interface (Fig. S3 B and Video 3), whereas it remains homogeneously distributed throughout the cell surface in nonactivating conditions (Fig. S3 A and Video 4). Thus, Fc receptors are recruited into the interface from around the cell surface.

To determine whether or not rings of the two FcγRs colocalized, cells were seeded onto nonactivating PLL– or activating hIgG–coated slides for 10 or 30 min and then fixed and stained with anti–FcγRI-AF488 and anti–FcγRII-AF647. Dual-color dSTORM revealed that FcγRI and FcγRII segregated at the nanometer scale in nonactivating conditions, as well as after 10 min of activation by hIgG (Fig. 5 B). CBC analysis (Fig. 5 C) and the mean NND of paired single-molecule localizations (Fig. 5 D) confirmed that FcγRI and FcγRII are segregated in nonactivated cells or cells activated for 10 min. The mode of NND between nanoclusters of the two receptors was >110 nm in each of these conditions (Fig. 5 E). However, after 30 min of activation, when both receptors form concentric rings, FcγRI and FcγRII were colocalized (Fig. 5 B), indicated by the CBC histogram distribution toward +1 (Fig. 5 C), the mean NND between localizations of 32 ± 16 nm (Fig. 5 D), and the mode of NND between nanoclusters of 61 ± 10 nm (Fig. 5 E). Thus, the assembly of rings of Fc receptor brings FcγRI and FcγRII together on a nanometer scale.

To establish whether or not ligation of Fc receptors or cellular activation in general causes segregation of FcγRI from SIRPα, and the assembly of rings of FcγRs, the effect of two different hIgG isotypes, hIgG1 and hIgG2, were compared. The high-affinity Fc receptor FcγRI can be activated by hIgG1, but not hIgG2, whereas the low-affinity Fc receptor FcγRII can be activated by both isotypes (Bruhns et al., 2009). If cellular activation in general is responsible for these rearrangements, then FcγRI would segregate from SIRPα and form concentric rings on hIgG2-coated surfaces that activate macrophages through other FcγRs, but not FcγRI. In contrast, if these events were a consequence of specific ligation, then both segregation between FcγRI and SIRPα and reorganization of FcγRI into rings would only be observed when the Fc receptor is ligated by hIgG1.

**Figure 6.**
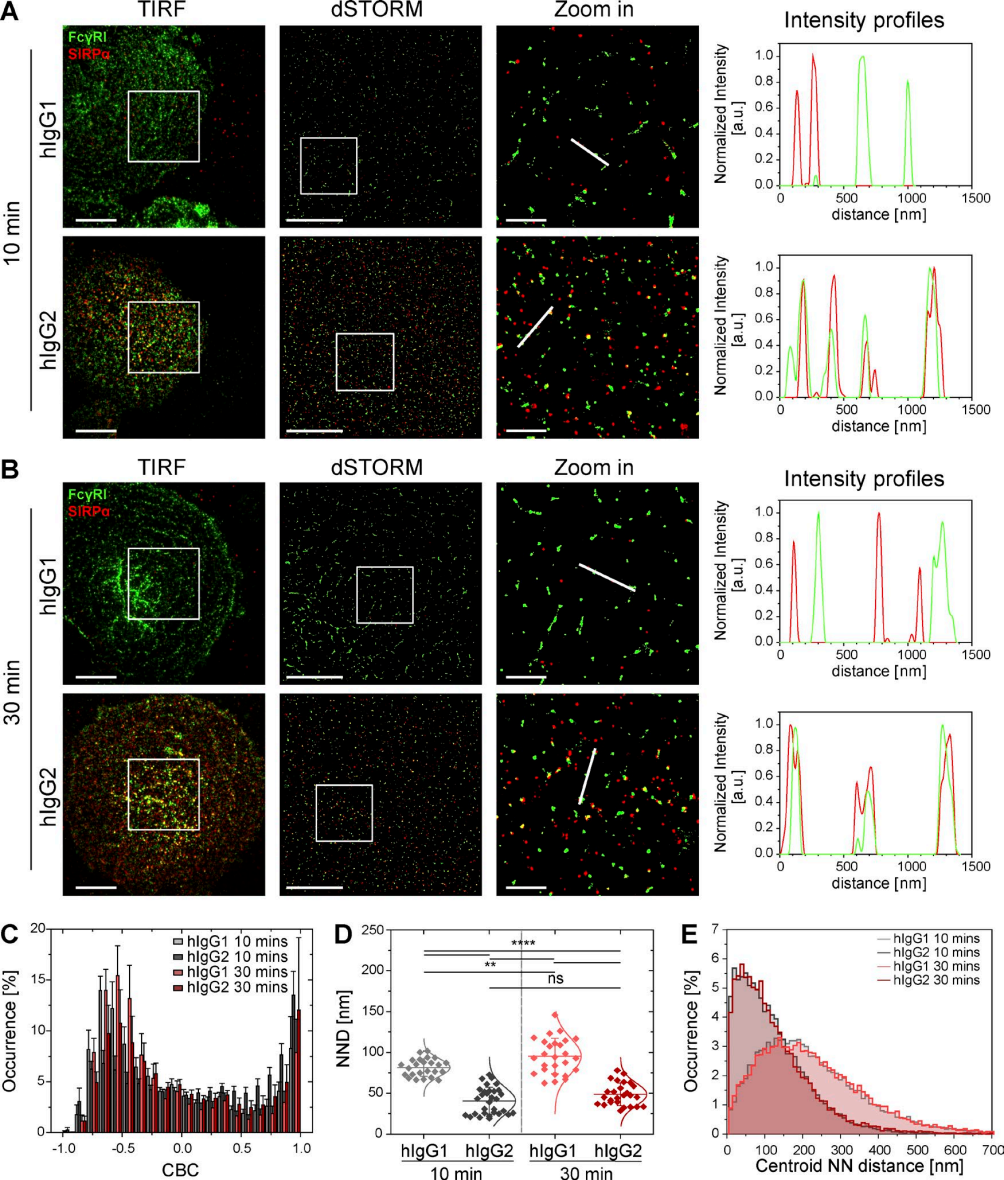
**Specific activation of FcγRI is required for its reorganization into concentric rings and segregation from SIRPα nanoclusters.** (A and B) TIRF (bars, 10 µm) and dSTORM (bars, 5 µm) images showing FcγRI (green) and SIRPα (red) at the surface of human macrophages incubated for 10 (A) or 30 min (B) on slides coated with hIgG1 or hIgG2 and stained with anti–FcγRI-AF488 and anti–SIRPα-AF647 mAbs. In each condition, regions outlined by the white squares (middle column) are shown enlarged (right column) with relative fluorescence intensity profiles along the white lines. Bars, 1 µm. (C) CBC histograms of the single-molecule distributions of the colocalization parameter for FcγRI and SIRPα in cells seeded onto hIgG1- or hIgG2-coated slides for 10 (light gray and dark gray, respectively) or 30 min (light red and dark red, respectively). Data are from a minimum of 30 cells from three independent donors. Bars represent mean ± SD. (D) NND analysis from data shown in C. Each symbol represents the median NND of all paired single-molecule localizations from one cell. Horizontal lines and error bars represent mean ± SD. ns, not significant; **, P < 0.01; ****, P < 0.0001; one-way ANOVA with Tukey’s post-hoc test. (E) Histogram distributions of the NND between the centroids of nanoclusters from one channel and the centroid of their nearest neighbor from the second channel (≥20,000 clusters from a minimum of 10 cells per condition). a.u., arbitrary units; NN, nearest neighbor; PC, positive control.

To test this, cells were plated onto hIgG1- or hIgG2-coated slides for 10 or 30 min, stained with anti–FcγRI-AF488 and anti–SIRPα-AF647 mAbs, and imaged. On hIgG1-coated slides, SIRPα and FcγRI nanoclusters were segregated and FcγRI reorganized into concentric rings (Fig. 6, A and B, top), resembling the results obtained with total hIgG. In contrast, when cells were plated onto hIgG2-coated slides, which activates cells via FcγRII, the FcγRI and SIRPα remained associated and FcγRI did not reorganize into concentric rings (Fig. 6, A and B, bottom). CBC analysis showed a shift in the CBC histogram toward −1 (Fig. 6 C) and a mean NND between localizations of 140 ± 31 nm (Fig. 6 D) on hIgG1, whereas the CBC histogram is shifted toward +1 upon stimulation with hIgG2 (Fig. 6 C) and the mean NND of paired single-molecule localizations was 61 ± 26 nm (Fig. 6 D). The mode of the centroid NND between nanoclusters was 181 ± 3 nm on hIgG1 and 66 ± 5 nm on hIgG2 (Fig. 6 E). These results were not time dependent, as the analysis was similar for 10 or 30 min of incubation (Fig. 6, B–E).

In contrast to FcγRI, FcγRII could reorganize into concentric rings after interacting with either hIgG1 or hIgG2 (Fig. S4 B). Reorganization of FcγRII occurred after 30 min of incubation, but not after 10 min (Fig. S4), consistent with stimulation with hIgG (Fig. 5 A), and when plated onto hIgG1-coated slides, FcγRII colocalized with FcγRI (Fig. S4 B). Thus, these data establish that the reorganization of FcγRI and FcγRII into a specific pattern of concentric rings occurs as a consequence of receptor ligation and not of cellular activation in general.

### The nanoscale organization of macrophage surfaces alters upon activation by membrane-bound IgG

**Figure 7.**
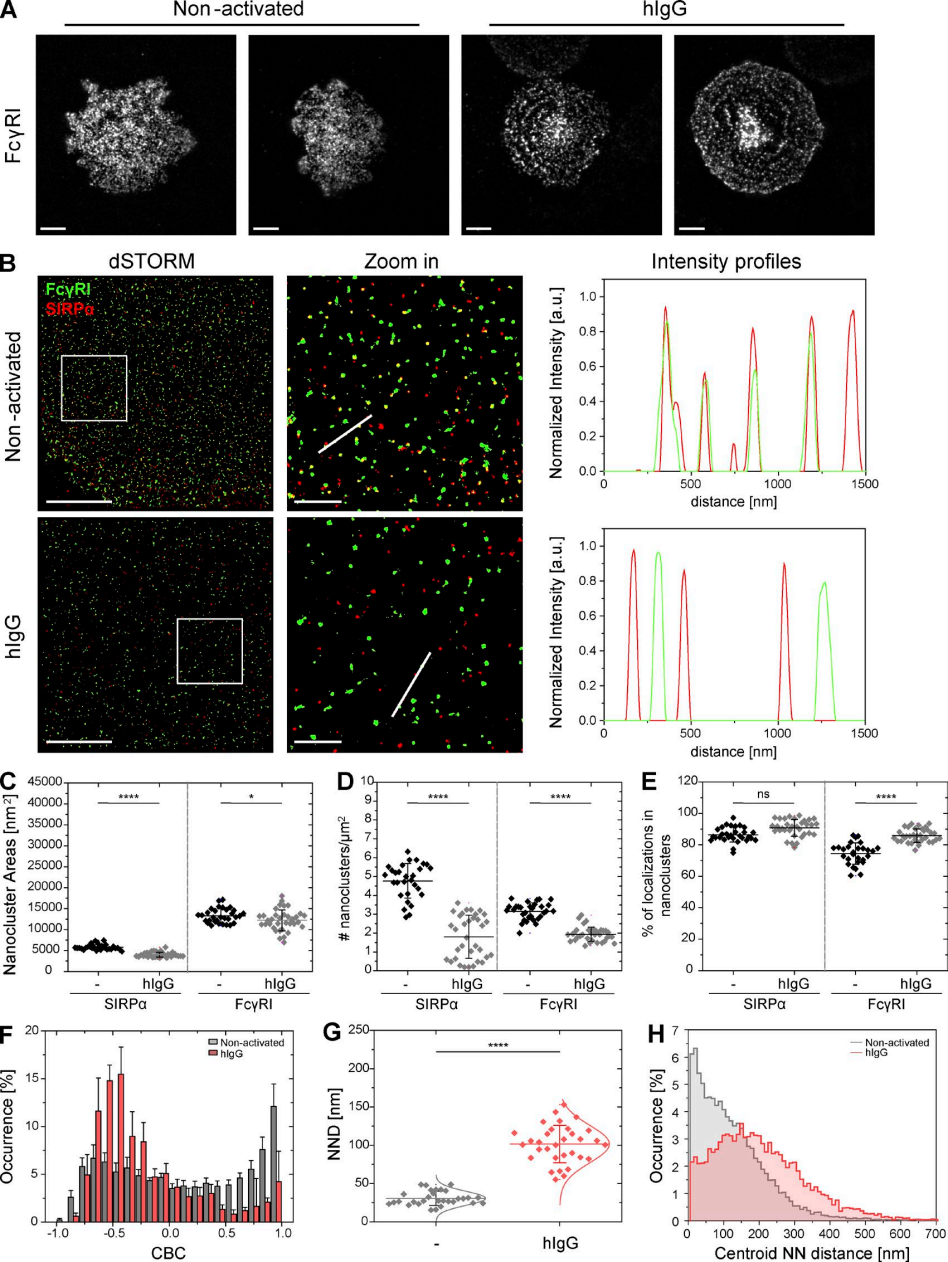
**Rearrangement of macrophage surface receptors triggered by mobile hIgG.** (A) TIRF images of FcγRI at the surface of human macrophages incubated for 10 min on SLBs loaded with streptavidin (nonactivating) or with streptavidin-hIgG (activating) and stained with a fluorescently labeled specific antibody. Two example images are shown for each condition. Bars, 10 µm. (B) dSTORM images of FcγRI (green) and SIRPα (red) at the surface of macrophages seeded as in A and stained with anti–FcγRI-AF488 and anti–SIRPα-AF647 mAbs. Bars, 5 µm. Regions outlined by the white squares are shown enlarged with relative fluorescence intensity profiles along the white lines. Bars, 1 µm. (C–E) Nanocluster areas (C), density (D), and percentage of localizations in nanoclusters (E) for SIRPα and FcγRI under nonactivating (black) or hIgG-activating (gray) conditions. Each symbol represents the median of several 5 × 5 µm regions from the same cell. Horizontal lines and error bars represent mean ± SD. Data are from a minimum of 30 cells from two independent experiments. ns, not significant; *, P < 0.05; ****, P < 0.0001; two-tailed *t* test assuming unequal variance. (F) CBC histograms of the single-molecule distributions of the colocalization parameter for SIRPα and FcγRI in cells seeded as in A. Data are from a minimum of 30 cells from two independent experiments. Bars represent mean ± SD. (G) NND analysis from data shown in F. Each symbol represents median NND of all paired single-molecule localizations from one cell. Horizontal lines and error bars represent mean ± SD. ****, P < 0.0001; two-tailed *t* test assuming unequal variance. (H) Histogram distributions of the NND between the centroids of nanoclusters from one channel and the centroid of their nearest neighbor from the second channel (≥10,000 clusters from a minimum of 10 cells per condition) from cells seeded onto control nonactivating (light gray) or hIgG-loaded activating (light red) SLBs.

To determine whether or not similar changes occur to the macrophage cell surface upon activation by mobile ligands, macrophages were seeded onto planar glass-supported lipid bilayers (SLBs) enriched with laterally mobile hIgG. After 10-min incubation, cells were fixed and stained with anti–FcγRI-AF488 and anti–SIRPα-AF647. For cells plated onto control SLBs, lacking hIgG, macrophage spreading was irregular and FcγRI nanoclusters were distributed across the cell surface (Fig. 7 A). In contrast, when cells were plated onto hIgG presented by SLBs, macrophages spread to a uniform radial morphology and FcγRI reorganized into concentric rings around a dense central accumulation (Fig. 7 A).

Quantitative analysis confirmed that SIRPα and FcγRI were organized in discrete nanoclusters on the surface of macrophages interacting with SLBs (Fig. 7, B–E), with characteristics similar to that for macrophages interacting with immobilized ligands on glass slides (Fig. 7, C–E; and Fig. 1, C–E). SIRPα assembled in slightly smaller nanoclusters (mean area of 5,800 ± 630 nm^2^, 43 ± 2 nm radius; Fig. 7 C), and nanoclusters were less abundant (mean of 4.6 ± 1.1 nanoclusters/µm^2^; Fig. 7 D) in comparison to cells interacting with glass slides. However, both the size and density of SIRPα nanoclusters decreased on hIgG-presenting SLBs (Fig. 7, C and D), as seen for immobilized hIgG. Most importantly, on control SLBs, SIRPα and FcγRI nanoclusters were localized in close proximity (distribution of the CBC histograms toward positive correlation coefficients, mean NND of paired single-localizations of 32 ± 12 nm, and a mode for the centroid NND of 23 ± 13 nm; Fig. 7, F–H) but segregated on hIgG-presenting SLBs (distribution of the CBC histograms toward negative correlation coefficients, mean NND of paired single-localizations of 95 ± 32 nm, and a mode for the centroid NND of 163 ± 3 nm; Fig. 7, F–H). Thus, nanoclusters of activating FcγRI segregate from inhibitory SIRPα and reorganize into concentric rings in a model of frustrated phagocytosis in which the activating ligand is embedded in lipid bilayers and is mobile.

### Ligation of SIRPα impairs the reorganization of surface FcγRI

**Figure 8.**
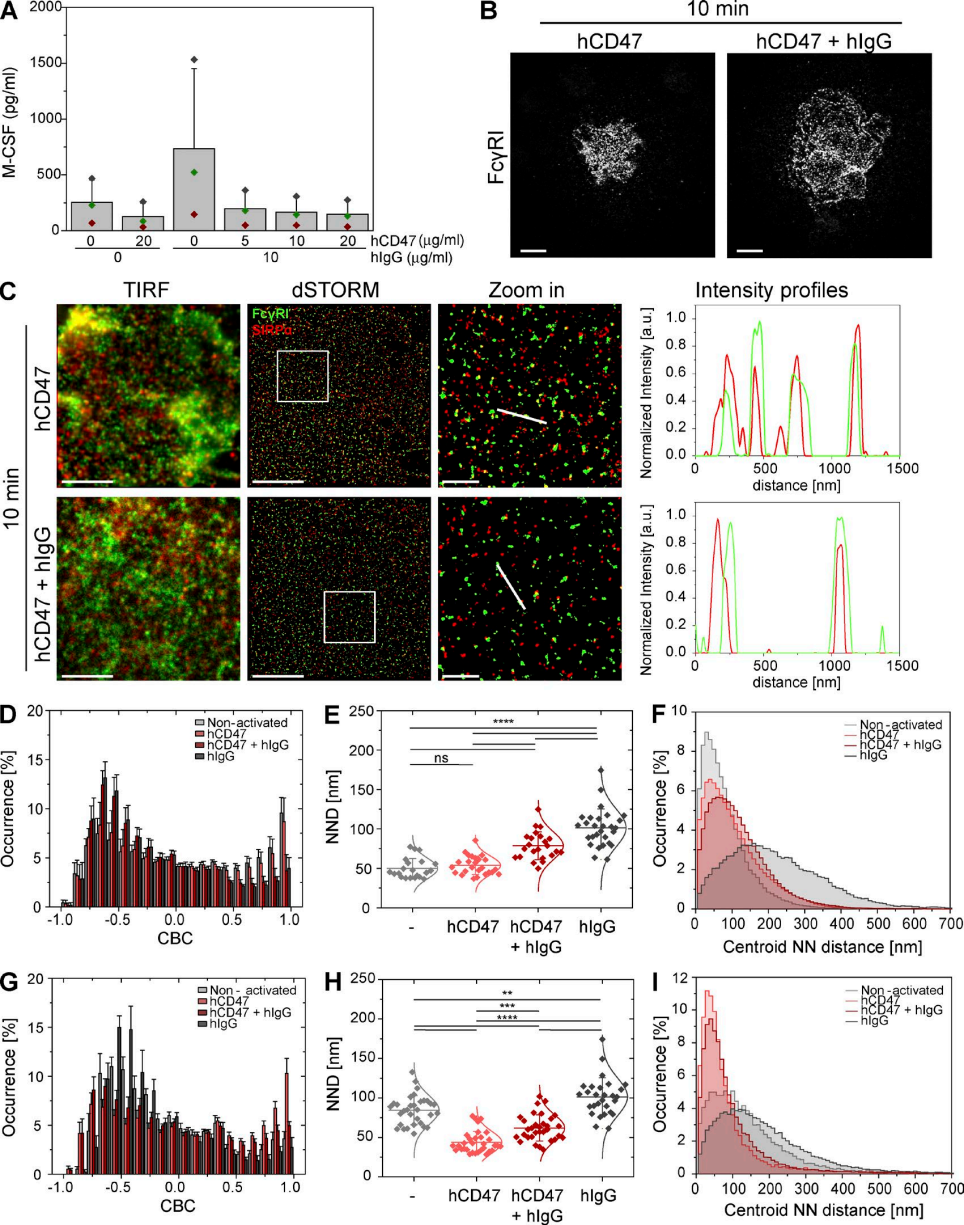
**Ligation of SIRPα impairs the reorganization of surface FcγRI.** (A) Human macrophages were incubated for 24 h in wells coated with PLL, 20 µg/ml of hCD47, or with increasing concentrations of hCD47 in the presence of 10 µg/ml of hIgG, as indicated. M-CSF release was assessed by ELISA. Bars represent mean ± SD from three donors. Each color represents one individual donor. (B) TIRF images of FcγRI at the surface of human macrophages incubated for 10 min on slides coated with hCD47 or hCD47 plus hIgG and stained with fluorescently labeled specific antibody. Bars, 10 µm. (C) TIRF and dSTORM images showing FcγRI (green) and SIRPα (red) at the surface of human macrophages incubated for 10 min on slides coated with hCD47 (top) or hCD47 plus hIgG (bottom) and stained with anti–FcγRI-AF488 and anti–SIRPα-AF647 mAbs. Bars, 5 µm. In each condition, regions outlined by the white squares (middle column) are shown enlarged (right column) with relative fluorescence intensity profiles along the white lines. Bars, 1 µm. (D and G) CBC histograms of the single-molecule distributions of the colocalization parameter for FcγRI and SIRPα (D) and for FcγRI and pSHP-1^Y536^ (G) in cells seeded onto slides coated with PLL (light gray), hCD47 (light red), hCD47 plus hIgG (dark red), or hIgG (dark gray) for 10 (D) or 5 min (G). Data are from a minimum of 30 cells from three independent donors. Bars represent mean ± SD. (E and H) NND analysis from data shown in D and G, respectively. Each symbol represents the median NND of all paired single-molecule localizations from one cell. Horizontal lines and error bars represent mean ± SD. ns, not significant; **, P < 0.01; ***, P < 0.001; ****, P < 0.0001; one-way ANOVA with Tukey’s post-hoc test. (F and I) Histogram distributions of the NND between the centroids of nanoclusters from one channel and the centroid of their nearest neighbor from the second channel (≥20,000 clusters from a minimum of 10 cells per condition). Graphs compare colocalization between FcγRI and SIRPα (F) and FCγRI and pSHP-1^Y536^ (I). a.u., arbitrary units; NN, nearest neighbor; PC, positive control.

We next asked whether the inhibitory signal from SIRPα could affect the micrometer- and nanometer-scale organization of these receptors. For this, cells were plated for 10 min onto slides coated with recombinant human CD47 protein (hCD47) to ligate SIRPα and hIgG to activate FcγRs, separately or in combination. Incubation of macrophages with hIgG led to release of macrophage colony-stimulating factor (M-CSF), measured by ELISA, which was reduced to basal levels in the presence of hCD47 (Fig. 8 A), confirming that hCD47 acted as a potent inhibitor of Fc receptor signals. In addition, upon coligation of SIRPα and Fc receptors, cells spread to an irregular shape (Fig. 8 B) and did not show the circular morphology characteristic of Fc receptor activation (Heiple et al., 1990; Cannon and Swanson, 1992). As before, stimulation with hIgG alone resulted in segregation of FcγRI and SIRPα (Fig. 8, D–F). However, segregation of these receptors caused by ligation of FcγRI could be abrogated by the simultaneous ligation of SIRPα with hCD47 (Fig. 8 C, bottom). CBC histograms did not reflect a strong level of inhibition by SIRPα (on account of the 50-nm search radius; Fig. 8 D), but the mean NND of paired single-molecule localizations (79 ± 18 nm; Fig. 8 E) and the mode of the centroid NND (89 ± 2 nm; Fig. 8 F) were significantly decreased upon coligation of inhibitory receptors (compared with ligation of Fc receptors alone; Fig. 8, E and F). In addition, FcγRI did not reorganize into concentric rings when SIRPα was coligated with the Fc receptors (Fig. 8 B). These results were reproduced after 30-min incubation, establishing that coligation of SIRPα does not merely delay the reorganization of cell surface nanoclusters (Fig. S5).

To further address if the nanoscale organization of the receptors is important for signal integration, we imaged FcγRI with SHP-1, the phosphatase recruited by SIRPα after activation with CD47, phosphorylated on tyrosine 536 (pSHP-1^Y536^), as a marker of phosphatase activity. Cells were plated for 5 min onto slides again coated with hCD47, to ligate SIRPα, and hIgG to activate FcγRs separately or in combination. Because SIRPα and FcγRI remain associated after ligation of SIRPα, recruitment of SHP-1 by the inhibitory receptor would bring the phosphatase to the close proximity of FcγRI. As such, ligation of SIRPα with CD47 resulted in the colocalization between pSHP-1^Y536^ and FcγRI nanoclusters (Fig. 8, G–I). Importantly, coligation of FcγRI and SIRPα also led to the colocalization between the activating receptor and pSHP-1^Y536^. The CBC histogram was shifted toward +1 (Fig. 8 G), and both the mean NND of paired single-molecule localizations (62 ± 16 nm; Fig. 8 H) and the mode of the centroid NND (56 ± 2 nm; Fig. 8 I) were similar to when SIRPα was ligated alone.

Overall, these data establish that signal integration between positive and negative signaling receptors impacts the nanoscale organization of the macrophage cell surface; colocalization of SIRPα and FcγRI nanoclusters correlates with cellular inhibition, whereas segregation of these nanoclusters correlates with activation.

### The actin cytoskeleton controls the proximity of SIRPα and FcγRI nanoclusters

**Figure 9.**
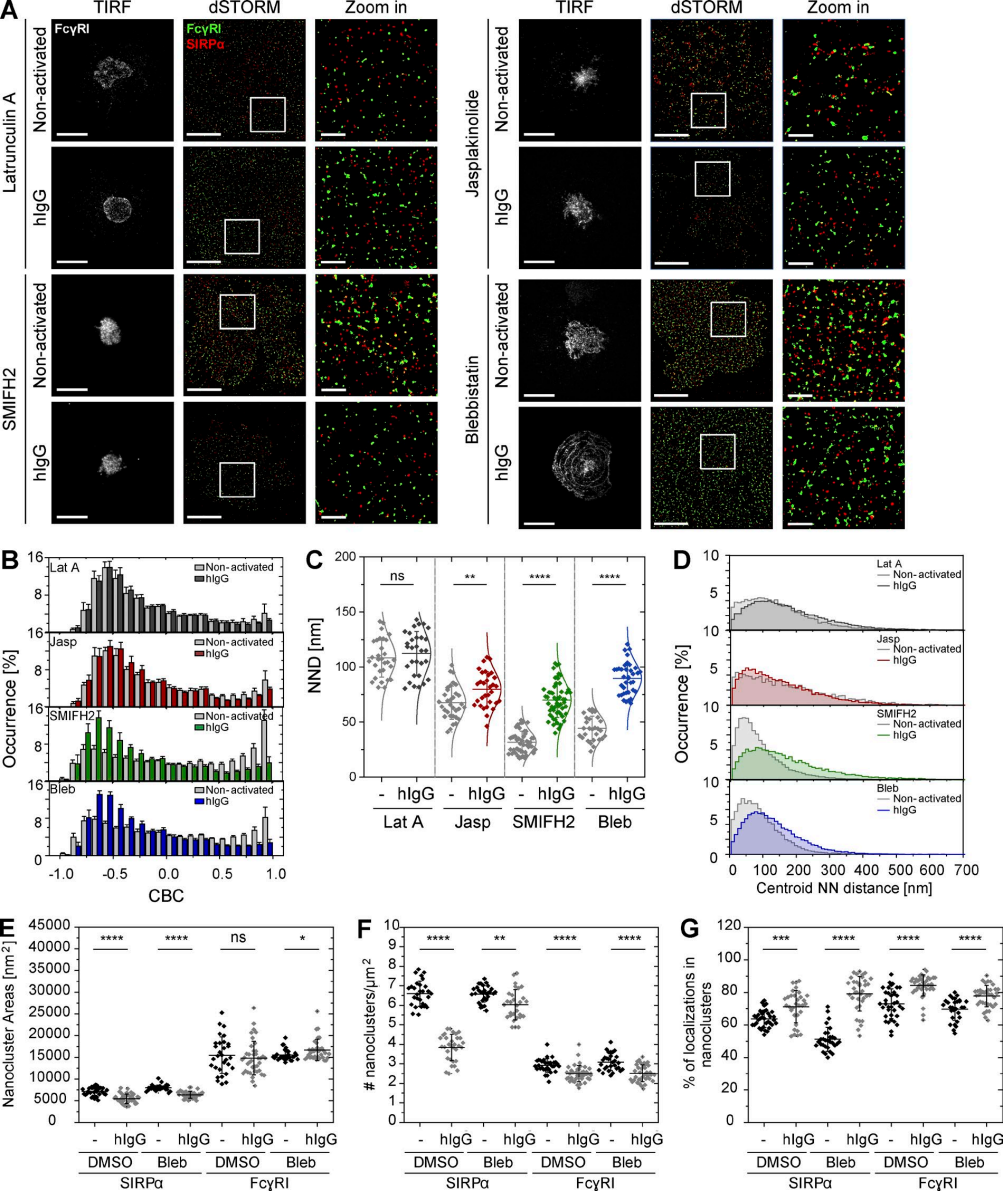
**Segregation and reorganization of FcγRI is dependent on the actin cytoskeleton and formins, but not myosin II.** (A) TIRF image of FcγRI (white; bars, 20 µm) and dSTORM images (bars, 5 µm) of FcγRI (green) and SIRPα (red) at the surface of human macrophages pretreated with 1 µM latrunculin A, 0.5 µM jasplakinolide, 10 µM blebbistatin or 10 µM SMIFH2. Cells were then seeded onto slides coated with PLL (nonactivated) or hIgG for 10 min, and stained with anti-FcγRI-AF488 and anti-SIRPα-AF647 mAbs. In each condition, regions outlined by the white squares (middle column) are shown enlarged (right column). Bars, 1 µm. (B) CBC histograms of the single-molecule distributions of the colocalization parameter for FcγRI and SIRPα in cells pretreated with drugs as indicated and seeded onto slides coated with PLL (gray) or hIgG (latrunculin A [Lat A], dark gray; jasplakinolide [Jasp], red; SMIFH2, green; or blebbistatin [Bleb], blue) for 10 min. Data are from a minimum of 30 cells per condition from three independent donors. Bars represent mean ± SD. (C) NND analysis from data shown in B. Each symbol represents the median NND of all paired single-molecule localizations from one cell. Horizontal lines and error bars represent mean ± SD. ns, not significant; **, P < 0.01; ****, P < 0.0001; two-tailed *t* test assuming unequal variance. (D) Histogram distributions of the NND between the centroids of nanoclusters from one channel and the centroid of their nearest neighbor from the second channel (≥20,000 clusters from a minimum of 10 cells per condition). (E–G) Nanocluster areas (E), density (F), and percentage of localizations in nanoclusters (G) for SIRPα and FcγRI under nonactivating (black) or hIgG-activating (gray) conditions after pretreatment of cells with blebbistatin or DMSO control. Each symbol represents the median of several 5 × 5 µm regions from the same cell. Horizontal lines and error bars represent mean ± SD. Data are from a minimum of 30 cells from three independent donors. ns, not significant; *, P < 0.05; **, P < 0.01; ***, P < 0.001; ****, P < 0.0001; two-tailed *t* test assuming unequal variance. NN, nearest neighbor.

One way in which proteins are organized at the cell surface is through interactions with the actin cytoskeleton (Lagrue et al., 2013; Mattila et al., 2013). To address the role of the cytoskeleton in the organization of SIRPα and FcγRI, macrophages were treated with pharmacological agents that interfere with the actin cytoskeleton. Treatment with latrunculin A, which disrupts the organization of filamentous actin by binding to, and sequestering, monomeric actin, induced the segregation of FcγRI and SIRPα in nonactivated cells (Fig. 9, A–D). This was indicated by the distribution of CBC histograms toward negative correlation coefficients (Fig. 9 B) and an increase in the NND of paired single-localizations (mean of 108 ± 17 nm; Fig. 9 C). In addition, the centroid NND between SIRPα and FcγRI nanoclusters increased (mode of 103 ± 3 nm; Fig. 9 D). Similarly, treatment with jasplakinolide, which stabilizes actin filaments, induced a small extent of segregation of nanoclusters in nonactivated conditions that became more pronounced upon stimulation with hIgG (Fig. 9, A–D). In both cases, the reorganization of FcγRI nanoclusters into concentric rings after stimulation was abolished (Fig. 9 A). In contrast, drugs which inhibit the activity of formins (SMIFH2; 1-(3-bromophenyl)-5-(2-furanylmethylene)dihydro-2-thioxo-4,6(1H,5H)-pyrimidinedione) or myosin II (blebbistatin) had little, if any, effect on the segregation of SIRPα and FcγRI nanoclusters (Fig. 9, A–D).

Assembly of the ring-shaped organization of FcγRI, however, was abolished by the inhibition of formins and not by inhibition of myosin II (Fig. 9 A), indicating that formins are important for this process. This also establishes that the assembly of Fc receptor into ring-shaped structures can be uncoupled from the segregation of SIRPα and FcγRI nanoclusters. Inhibition of myosin II by blebbistatin prevented the internalization of SIRPα at the interface to some extent (Fig. 9, E–G). Both the area (Fig. 9 E) and the density (Fig. 9 F) of SIRPα nanoclusters after hIgG stimulation was not as decreased as in DMSO-treated cells, suggesting that this process requires the activity of myosin II and that this too is independent of the segregation of SIRPα and FcγRI nanoclusters triggered upon activation. Together, these data establish that the constitutive association of FcγRI and SIRPα nanoclusters requires the actin cytoskeleton and that the mechanism underlying the segregation of receptors and the reorganization of FcγRI into concentric rings is dependent on the actin cytoskeleton and formins, but not myosin II.

### Segregation of FcγRI from SIRPα and reorganization of FcγRI into concentric rings is dependent on SFK signaling

**Figure 10.**
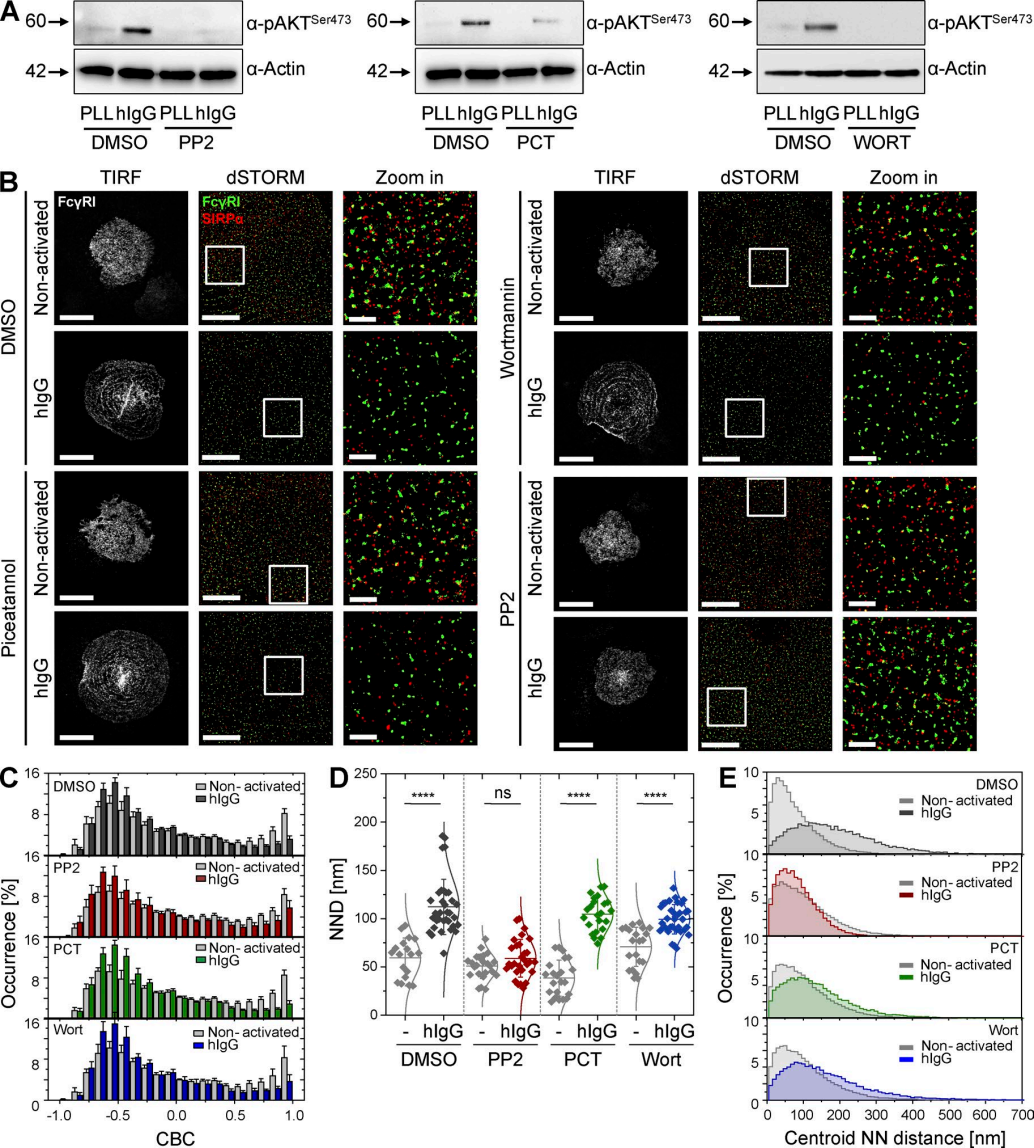
**Src-family kinase signaling, but not Syk or PI3K signaling, is indispensable for reorganization of macrophage surfaces.** (A) Immunoblots of phosphorylated AKT in nonactivated (PLL) or hIgG-activated human macrophages pretreated with vehicle (DMSO), as a control, 10 µM PP2 (left), 100 µM piceatannol (PCT; middle), or 1 µM wortmannin (Wort; right). Blots represent two independent experiments. (B) TIRF image of FcγRI (white; bars, 20 µm) and dSTORM images (bars, 5 µm) of FcγRI (green) and SIRPα (red) at the surface of human macrophages incubated with vehicle (DMSO), PP2, PCT, or Wort, pretreated as in A. Cells were then seeded onto slides coated with PLL (nonactivated) or hIgG for 10 min and stained with anti–FcγRI-AF488 and anti–SIRPα-AF647 mAbs. In each condition, regions outlined by the white squares (middle column) are shown enlarged (right column). Bars, 1 µm. (C) CBC histograms for FcγRI and SIRPα in cells pretreated as in A and seeded onto slides coated with PLL (gray) or hIgG (DMSO, dark gray; PP2, red; PCT, green; and Wort, blue) for 10 min, as indicated. Data are from a minimum of 30 cells from three independent donors. Bars show mean ± SD. (D) NND analysis from data shown in C. Each symbol represents the median NND of all paired single-molecule localizations from one cell. Horizontal lines and error bars represent mean ± SD. ns, not significant; ****, P < 0.0001; one-way ANOVA with Tukey’s post-hoc test. (E) Histogram distributions of the NND between the centroids of nanoclusters from one channel and the centroid of their nearest neighbor from the second channel (≥20,000 clusters from a minimum of 10 cells per condition).

To better understand the mechanism of SIRPα and FcγRI segregation and the reorganization of the FcγR into concentric rings, we next set out to dissect the signaling pathways involved using drugs known to target specific components of the phagocytic machinery. The effectiveness of PP2 (an SFK inhibitor), piceatannol (an Syk inhibitor), and wortmannin (a phosphoinositide 3-kinase [PI3K] inhibitor) was verified by immunoblotting with an antibody for phosphorylated AKT, a downstream target of both SFK and PI3K signaling (Fig. 10 A).

Inhibition of Syk or PI3K had no effect on the segregation of SIRPα and FcγRI or on the reorganization of FcγRI into concentric rings upon activation of cells with hIgG (Fig. 10 B). Both CBC analysis and centroid NND analysis showed equivalent segregation of receptors in control-treated cells or cells treated with either of the two drugs (Fig. 10, C–E). In contrast, when SFKs were inhibited, SIRPα and FcγRI remained colocalized after stimulation of cells with hIgG (Fig. 10, B and C) with a mean NND between localizations (62 ± 22 nm) and a mode for the centroid NND (71 ± 1 nm) similar to DMSO-treated control cells in nonactivating conditions (mean NND between localizations of 62 ± 23 nm and mode of the centroid NND of 52 ± 3 nm; Fig. 10, D and E). In addition, the assembly of Fc receptor rings was abrogated by PP2 (Fig. 10 B). Thus, membrane proximal signaling by SFKs is important for the nanometer- and micrometer-scale reorganization of the macrophage cell surface.

## Discussion

It is well established that immune cell receptors and ligands are organized into micrometer- and submicron-scale domains at cell surfaces and immune synapses (Bunnell et al., 2002; Davis and Dustin, 2004; Harwood and Batista, 2008; Orange, 2008). Recently, however, the development of superresolution microscopy techniques has extended this view by providing evidence that many proteins are organized in plasma membrane domains on a nanometer scale (Garcia-Parajo et al., 2014).

A previous study indicated that FcγRII is constitutively expressed as monomers and upon ligation increases its lateral mobility and clustering (Jaumouillé et al., 2014). Here, exploiting recent developments in superresolution microscopy, we revise this model of the macrophage cell surface by establishing that the activating receptors FcγRI and FcγRII, and the inhibitory receptor to prevent phagocytosis of self, SIRPα, are constitutively organized in discrete nanometer-scale domains at the surface of primary human macrophages, with SIRPα forming smaller but more numerous nanoclusters than the Fc receptors.

In nonactivated cells, nanoclusters of FcγRI, but not FcγRII, are associated with nanoclusters of SIRPα. Ligation of SIRPα recruits the phosphatase SHP-1, which likely acts locally. Thus, the proximity between FcγRI and SIRPα is likely to be important for SIRPα-mediated inhibition of the Fc receptor signaling. Disruption of filamentous actin organization induced the segregation between SIRPα and FcγRI even in nonactivating conditions. This suggests that the constitutive association between the two receptors is regulated to at least some extent by the actin cytoskeleton.

For T cells and B cells, activating signals are concurrent with the segregation of phosphatase activity from kinase activity (Chang et al., 2016). Here, stimulation with hIgG induced segregation of FcγRI and SIRPα nanoclusters. This likely hinders the ability of SIRPα signaling to suppress FcγRI signaling and thereby provides a positive feedback to amplify or sustain cellular activation. In contrast, coligation of SIRPα with CD47 abrogated the segregation of SIRPα and FcγRI nanoclusters and promoted the recruitment of pSHP-1^Y536^ to the proximity of FcγRI nanoclusters, thereby helping prevent cellular activation. Previous imaging has not detected these nanoscale rearrangements at the cell surface, because they could not be resolved by standard light microscopy.

Interaction with IgG also induced the reorganization of FcγRs at a micrometer scale. After cross-linking, FcγRs were recruited to the frustrated phagocytic synapse, where they formed periodically spaced concentric rings. Live imaging of FcγRI showed that the reorganization of the receptor into concentric rings is coincident with cell spreading on activating surfaces. An expanding integrin wave is known to extend beyond the perimeter of the receptor–ligand engagement zone and facilitate the zippering of FcγRs onto the target (Freeman et al., 2016). It is possible that this actin-tethered integrin wave may play a role in positioning FcγRs in concentric rings, such that the rings of Fc receptor mark out the spaced teeth of a phagocytic zipper.

Mechanistically, the reorganization of FcγRI into concentric rings, as well as the segregation of SIRPα and FcγRI nanoclusters, is dependent on the actin cytoskeleton. Assembly of the ring-shaped structure of FcγRI is also dependent on formins but independent of myosin II activity. In contrast, myosin II activity is important for the internalization of SIRPα triggered by FcγRI ligation. Moreover, inhibition of SFKs, which are essential for an efficient phagocytic response, prevented these rearrangements of the macrophage cell surface. Thus, impairing the actin cytoskeleton or blocking the phagocytic signal, either by inhibiting the activating signal with pharmacological drugs or by ligating the inhibitory receptor, hindered both the segregation between FcγRI and SIRPα and the reorganization of FcγRI into concentric rings, pointing to these behaviors being an important feature of the phagocytic response.

In summary, the high-affinity Fc receptor FcγRI is kept in close proximity to the inhibitory receptor SIRPα by the actin cytoskeleton in nonactivated macrophages. Upon ligation of FcγRI, nanoclusters of FcγRI segregate from nanoclusters of SIRPα. This occurs concurrently with a micrometer-scale reorganization of activating Fc receptors into concentric rings, dependent on SFK signaling. Coligation of SIRPα abrogates this segregation of nanoclusters, promotes the recruitment of pSHP-1^Y536^ to the proximity of FcγRI, and prevents the assembly of Fc receptor rings. Altogether, these data reveal an unexpected nanometer- and micrometer-scale rearrangement of the macrophage cell surface concurrent with signal integration.

## Materials and methods

### Generation of macrophages

Peripheral blood from healthy donors was acquired from the National Health Service blood service under ethics license REC 05/Q0401/108 (University of Manchester). Peripheral blood mononuclear cells were isolated by density gradient centrifugation (Ficoll-Paque Plus; Amersham Pharmacia Biotech). Human monocyte-derived macrophages were derived as described previously (Davies and Gordon, 2005). In brief, CD14^+^ cells were isolated by positive selection from peripheral blood mononuclear cells using magnetic beads (CD14 MicroBeads; Miltenyi Biotec) and cultured at 10^6^ cells/ml in serum-free media (X-Vivo 10; Lonza) supplemented with 1% human serum (Sigma-Aldrich). After 24 h, monocytes were washed with PBS (Sigma-Aldrich) to remove nonadherent cells and cultured in X-Vivo media with 1% human serum. After 3 d of incubation, adherent cells were washed with PBS and cultured in standard DMEM-based media (Sigma-Aldrich) supplemented with 10% FBS (Invitrogen), 1% penicillin and streptomycin (Gibco), 1% l-glutamine (Gibco), and 1% Hepes (Sigma-Aldrich) for 6 days to generate monocyte-derived macrophages, phenotyped to be CD14^+^, CD11a^+^, CD3^−^, CD56^−^, and CD19^−^. Cells were washed with PBS, and media was replaced every 3 d.

### Flow cytometry

To assess surface expression of SIRPα and FcγRI, cells were washed and blocked with 2% FBS/PBS for 30 min at 4°C and then stained with Zombie Aqua viability dye (BioLegend), anti–CD14 mAb (clone 61D3; eBioscience) conjugated with FITC and anti–SIRPα mAb (clone SE5A5; BioLegend) or anti-FcγRI (clone 10.1; BioLegend), respectively, conjugated with Allophycocyanin (APC) or isotype-matched control mAbs (mouse IgG1 isotype control, clone MOPC-21; BioLegend; conjugated with FITC or APC) for 30 min at 4°C. To assess surface expression of FcγRII, cells were washed and blocked as before and then stained with Zombie Aqua viability dye, anti–CD14 mAb (clone 61D3; eBioscience) conjugated with APC, anti–FcγRII mAb (clone FLI8.26; BD), or isotype-matched control mAb (mouse IgG2b isotype control, clone MPC-11; BD) for 30 min at 4°C, washed twice with 2% FBS/PBS, and incubated with fluorescently labeled anti–mouse IgG2b secondary antibody (Invitrogen) conjugated with AF488 for 30 min at 4°C. Finally, cells were washed in 2% FBS/PBS, fixed in 2% PFA/PBS, assessed by BD FACS Canto II flow cytometer (BD), and analyzed (FlowJo_V10 software).

For phenotyping, monocyte-derived macrophages were stained with anti–CD14 mAb (clone 61D3; eBioscience), anti–CD11a mAb (clone HI111; BD), anti–CD3 mAb (clone UCHT1; BioLegend), anti–CD56 mAb (clone HCD56; BioLegend), and anti–CD19 mAb (clone HIB19; BioLegend) or isotype-matched control mAb (mouse IgG1 isotype control, clone MOPC-21, conjugated with APC or phycoerythrin [BioLegend] or FITC [BD]). Anti–CD14 and anti–CD11a mAbs are conjugated with APC and FITC, respectively, whereas anti–CD3, anti–CD56, and anti–CD19 mAbs are conjugated with phycoerythrin.

### ELISA

Primary monocyte-derived macrophages were incubated on chambered glass coverslips coated with PLL, human CD47-Fc, or human IgG, as indicated, at 37°C for 24 h. Cell supernatants were recovered and centrifuged at 350 *g* for 10 min at RT to remove cell debris. M-CSF production was quantified in the supernatants by sandwich ELISA (DuoSet ELISA; R&D Systems), according to manufacturer’s instructions. The plates were developed with TMB ELISA substrate (Sigma-Aldrich), and the reaction was stopped with 1 N H_2_SO_4_. Absorbance was measured at 450 nm using a 570-nm reference line.

### Drug treatments

Primary human macrophages were pretreated with 10 µM of either the SFK inhibitor PP2 (Sigma-Aldrich) or the myosin II inhibitor blebbistatin (Sigma-Aldrich), with 100 µM of the Syk kinase inhibitor piceatannol (Sigma-Aldrich), or with 0.5 µM jasplakinolide (Sigma-Aldrich) for 30 min, or with 1 µM of either latrunculin A (EMD Millipore) or the PI3K inhibitor wortmannin (Sigma-Aldrich), or with 10 µM of the formin inhibitor SMIFH2 (Sigma-Aldrich) for 10 min, in PBS at 37°C. As a control, cells were incubated with DMSO. After incubation, cells were resuspended in culture medium and plated onto coverslips under nonactivating or activating conditions for 10 min, as indicated, before being fixed and stained for imaging.

### Immunoblotting

Primary human macrophages were pretreated as described in the previous paragraph and plated onto PLL- or hIgG-coated slides for 10 min at 37°C. After incubation, adherent cells were rinsed twice in ice-cold PBS and disrupted with ice-cold lysis buffer containing 10 mM Tris-HCl, pH 7.4, 100 mM NaCl, 1mM NaF, 1 mM orthovanadate, 0.5% NP-40, and protease inhibitors (cOmplete EDTA-free protease inhibitors; Roche). Lysates were cleared by centrifugation and reduced in Laemmli buffer, resolved by SDS-PAGE, transferred to nitrocellulose membrane, and immunoblotted with anti–phospho-AKT Ser 473 (clone 193H12; Cell Signaling Technology) and anti-actin (Sigma-Aldrich) antibodies.

### Sample preparation for imaging

Chambered glass coverslips (#1.5 Lab-Tek II; Nunc) were coated with 0.01% PLL (Sigma-Aldrich) and used for imaging of unstimulated cells or coated with 10 µg/ml hCD47-Fc (R&D Systems), 10 µg/ml hIgG, 10 µg/ml hIgG1, or 10 µg/ml hIgG2 (freshly resuspended in 150 mM NaCl; all from Sigma-Aldrich) in PBS at 4°C (overnight), as indicated, for stimulation of cells. Cells were allowed to settle on the slides for 5, 10, or 30 min at 37°C, fixed with 4% PFA/PBS for 15 min at RT, and washed three times in PBS. In other experiments, we confirmed that similar results were obtained when cells were fixed with 4% PFA/PBS for 1 h or with 4% PFA/0.2% glutaraldehyde for 30 min. Samples were blocked in 3% BSA/PBS for 1 h at RT followed by incubation with the appropriate fluorescently labeled mAbs, diluted in 3% BSA/PBS for 1 h at RT. Whenever intracellular staining was required cells were first permeabilized and blocked with 3% BSA/0.2% Triton X-100/PBS at RT for 1 h before incubation with the antibody. Samples were then washed, postfixed with 4% PFA/PBS for 5 min at RT, and imaged. Primary monoclonal antibodies used for microscopy were anti-SIRPα (clone 4C7; AbD Serotec) conjugated in-house with AF647 (Invitrogen), anti–FcγRI-AF488 (clone 10.1; BioLegend), anti-FcγRII (clone FLI8.26; BD) conjugated in-house with Atto488 (Invitrogen) or AF647, and anti–PTPN6(Tyr536)-AF647 (Bioss). All in-house–labeled antibodies had six or seven dyes per antibody.

### SLBs

Preparation of liposomes and planar bilayer formation are described in detail elsewhere (Dustin et al., 2007). In brief, for coupling of streptavidin-conjugated hIgG, prepared using a Streptavidin Conjugation kit (Abcam) according to the manufacturer’s instructions, 2 mol% DOPE-cap-Biotin in 1,2-dioleoyl-sn-glycero-3-phosphocholine were deposited onto clean glass coverslip of the flow chamber (sticky-Slide VI 0.4; Ibidi). As a control, planar bilayers were coupled with streptavidin-AF647 (Molecular Probes). Lipid droplets were trapped by overlaying glass coverslips cleaned using peroxidated H_2_SO_4_. Chambers were flooded with Hepes buffered saline supplemented with 0.1% BSA and flushed to remove excess liposomes, leaving deposited 1,2-dioleoyl-sn-glycero-3-phosphocholine bilayers containing 2 mol% DOPE-cap-Biotin. Bilayers were uniformly fluid as measured by photobleaching/recovery. After blocking for 30 min with Hepes buffered saline supplemented with 2% BSA, fluorescently labeled (or unlabeled) hIgG was incubated on bilayers for 30 min. Protein concentrations required to achieve desired densities on bilayers were calculated from calibration curves constructed from flow-cytometric measurements of bilayer-associated fluorescence of attached proteins on bilayers form on glass beads, compared with reference beads containing known numbers of the appropriate fluorophore (Bangs Laboratories). All lipids were purchased from Avanti Polar Lipids, Inc. Cells were allowed to settle and form contacts with the bilayer for 10 min before being fixed with 4% PFA for 15 min, labeled, and imaged by dSTORM.

### Confocal imaging

Confocal imaging (TCS SP8; Leica Biosystems) was performed using a 100× 1.4-NA oil-immersion objective. Cells were plated onto PLL- or hIgG-coated slides for 10 min and then fixed and stained with NucBlue Live Cell Stain (Invitrogen), the membrane dye Vybrant DiD (1,1-Dioctadecyl-3,3,3,3-tetramethylindodicarbocyanine; Invitrogen), and anti–FcγRI-AF488 mAb (clone 10.1; BioLegend). Images were taken with a frame rate of 600 Hz and 250 nm *z*-stepping. ImageJ (National Institutes of Health) was used for 3D rendering.

### TIRF and live-cell imaging

TIRF and live-cell imaging (N-STORM; Nikon) were performed using a 100× 1.49-NA oil-immersion objective. For TIRF imaging, cells were plated onto PLL- or hIgG-coated slides for 10 or 30 min and then fixed and stained with anti–FcγRI-AF488 (clone 10.1, Biolegend) or anti-FcγRII (clone FLI8.26; BD) conjugated in-house with Atto488 (Invitrogen) as described previously. For live-cell imaging, cells were stained with anti-FcγRI-AF488 mAb as previously described for FACS experiments and imaged immediately as they landed onto PLL- or hIgG-coated slides. The emitted fluorescence was collected by the objective onto an electron-multiplying charge-coupled device camera (IXON Ultra 897; Andor Technology). The frame rates used in the experiments were 100 ms (for PLL) or 200 ms (for hIgG) per frame for a maximum of 10 min. A focus lock system was used to keep the sample in focal plane.

### dSTORM imaging

dSTORM imaging (SR GSD; Leica Biosystems) was performed using a 160× 1.43-NA oil-immersion objective in TIRF mode. Dual-color dSTORM imaging was performed with primary antibodies directly conjugated with AF647 and AF488 acquired in sequential manner. First, 642-nm laser light was used for exciting the AF647 dye and switching it to the dark state. Second, 488-nm laser light was used for exciting the AF488 dye and switching it to the dark state. An additional 405-nm laser light was used for reactivating the AF647 and AF488 fluorescence. The emitted light from both dyes was collected by the same objective and imaged onto the electron-multiplying charge-coupled device camera at a frame rate of 10 ms per frame. A maximum of 5,000 frames per condition were acquired. For each receptor, the specificity of the labeling was confirmed by staining cells with isotype-matched control antibodies (Fig. S3). These controls showed a negligible level of nonspecific binding (no more than 3% of the total number of localizations per cell).

### dSTORM data analysis

Because dual-color dSTORM imaging is performed in sequential mode by using two different optical detection paths (dichroic and emission filters are different), an image registration is required to generate the final two-color dSTORM image (Bates et al., 2012; Bálint et al., 2013). Therefore, fiducial markers (TetraSpek Fluorescent Microspheres; Invitrogen) of 100 nm, which were visible in both 488-nm and 647-nm channels, were used to align the 488-nm channel to 647-nm channel. The images of the beads in both channels were used to calculate a polynomial transformation function that maps the 488-nm channel onto the 647-nm channel, using the MultiStackReg plug-in of ImageJ to account for differences in magnification and rotation, for example. The transformation was applied to each frame of the 488-nm channel. dSTORM images were analyzed and rendered as previously described (Bates et al., 2007; Huang et al., 2008) using custom-written software (Insight3, provided by B. Huang, University of California, San Francisco). In brief, peaks in single-molecule images were identified based on a threshold and fit to a simple Gaussian to determine the *x* and *y* positions. Only localizations with photon count >400 photons were included, and localizations that appeared within one pixel in five consecutive frames were merged together and fitted as one localization. The final images were rendered by representing the *x* and *y* positions of the localizations as a Gaussian with a width that corresponds to the determined localization precision. Sample drift during acquisition was calculated and subtracted by reconstructing dSTORM images from subsets of frames (500 frames) and correlating these images to a reference frame (the initial time segment).

Quantitative cluster analysis was based on Ripley’s *K* function (Ripley, 1977) and univariate Getis and Franklin’s local point pattern analysis (Getis and Franklin, 1987; Perry, 2004). The *x* and *y* coordinate list of localizations was used and multiple regions of 5 × 5 µm were selected for each cell, giving the median value per cell. Spatial pattern analysis using Ripley’s *K* function was performed with SpPack (Perry, 2004). Quantitative color scale cluster maps based on univariate Getis and Franklin’s local point pattern analysis method were generated using a custom MATLAB script as described previously (Owen et al., 2010) with a sampling radius of 50 nm. 2D pseudocolor density maps were created by interpolating a surface plot with L(50) as the z axis on a grid of resolution 5 nm. Binary maps, generated from density maps, were used to measure cluster sizes and the number of clusters per square millimeter in ImageJ by using the particle analysis function. Varying label density analysis was performed as described previously (Baumgart et al., 2016).

### CBC analysis

CBC mediated analysis between two receptors was performed using an ImageJ plug-in (Ovesný et al., 2014) based on an algorithm described previously (Malkusch et al., 2012). To assess the correlation function for each localization, the x-y coordinate list from 488-nm and 647-nm dSTORM channels was used. For each localization from the 647-nm channel, the correlation function to each localization from the 488-nm channel was calculated. This parameter can vary from −1 (perfectly segregated) to 0 (uncorrelated distributions) to +1 (perfectly colocalized). The correlation coefficients were plotted as a histogram of occurrences with a 0.1 binning. The NND between each localization from the 647-nm channel and its closest localization from the 488-nm channel was measured and plotted as the median NND between localizations per cell.

To assess protein cluster colocalization, centroid NND were calculated using an ImageJ plug-in as described in the preceeding paragraph. Dual-color dSTORM images were converted into binary maps, and the *x* and *y* coordinates of cluster centroids were identified in each image using the particle analysis function in ImageJ. The NND from the centroid of a cluster in the 488-nm channel to the closest centroid of a cluster in the 647-nm channel was measured and plotted as a histogram of occurrences with a 10-nm binning. Experimental data were compared against randomized equivalents where the red (647-nm) channel images were randomly assigned new centroid coordinates within a region of interest delineating the cell boundary. The mode of the histograms was determined by fitting the distribution to a Gaussian function.

### Statistical analysis

Samples were tested for normality with a Kolmogorov–Smirnov test. The statistical significance of differences between two datasets was assessed by a two-tailed *t* test assuming unequal variance; multiple comparisons were made with one-way analysis of variance with Tukey’s post-hoc test. All statistical analysis was performed using Origin software (OriginLab).

### Online supplemental material

Fig. S1 shows the phenotyping of macrophages and the analysis of SIRPα, FcγRI, and FcγRII surface expression by flow cytometry. Fig. S2 shows the nanometer-scale organization of SIRPα and FcγRI at 30 min of incubation. Fig. S3 shows the reorganization of FcγRI into concentric rings at the phagocytic synapse by confocal microscopy and isotype-matched control staining for all antibodies used in this study. Fig. S4 shows that the engagement of FcγRs is required for their reorganization into concentric rings. Fig. S5 shows that SIRPα inhibition of FcγRI segregation and reorganization into concentric rings persists at 30 min of activation. Video 1 shows live TIRF imaging of the distribution of FcγRI at the surface of macrophages during cell spreading under nonactivating conditions. Video 2 shows live TIRF imaging of the formation of FcγRI concentric rings at the surface of macrophages during cell spreading under activating conditions. Video 3 shows a representative confocal Z-stack and 3D projection of the distribution of FcγRI at the surface of macrophages under activating conditions. Video 4 shows a representative confocal Z-stack and 3D projection of the distribution of FcγRI at the surface of macrophages under nonactivating conditions.

## Supplementary Material

Supplemental Materials (PDF)

Video 1

Video 2

Video 3

Video 4
