# Supplementary material for: Membrane nanoclusters of FcγRI segregate from inhibitory SIRPα upon activation of human macrophages
Source: J Cell Biol. 2017 Apr 3;216(4):1123–41. doi: 10.1083/jcb.201608094 (PMC5379948; doi:10.1083/jcb.201608094)
Supplement: Supplemental Materials (PDF) [file JCB_201608094_sm.pdf]

Lopes et al., https://doi.org/10.1083/jcb.201608094

THE JOURNAL OF CELL BIOLOGY

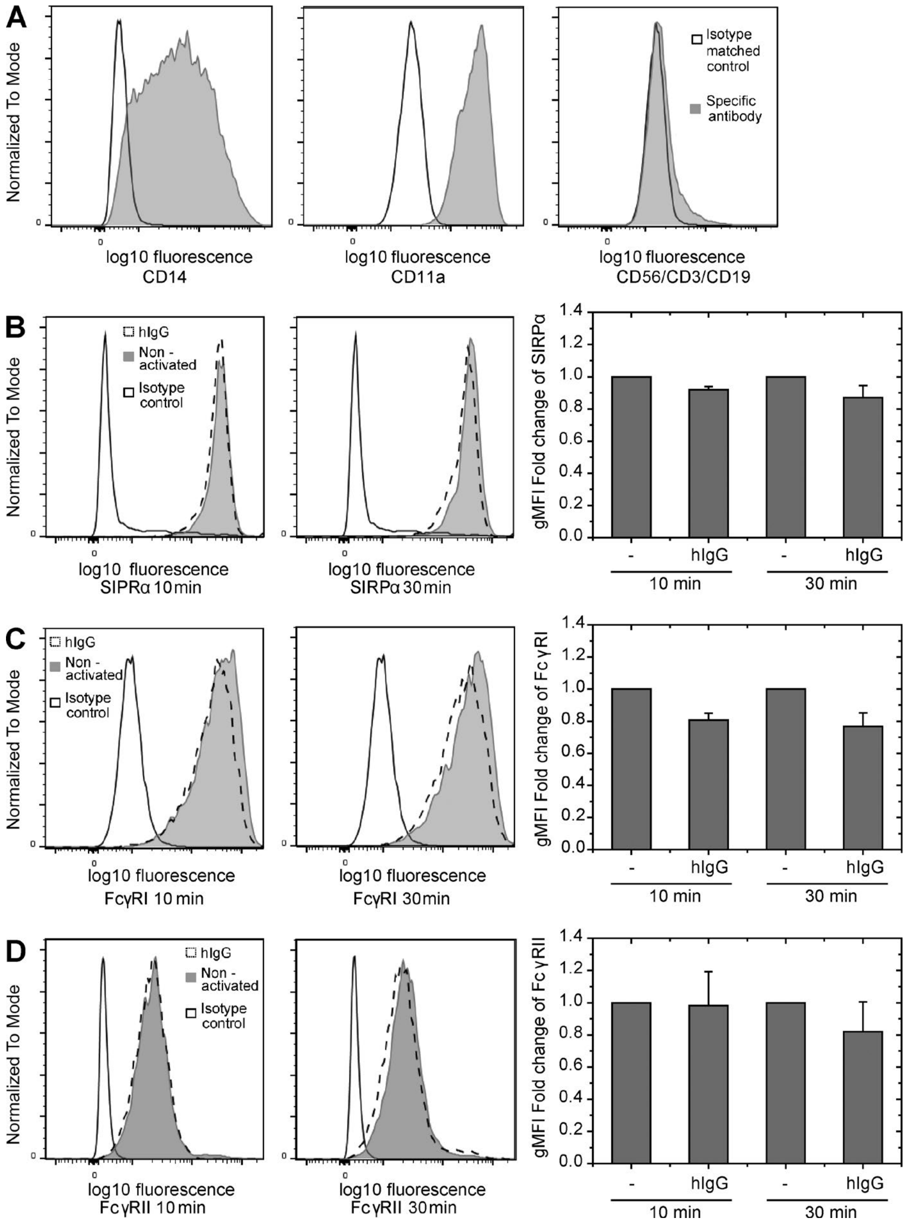

Figure S1. **Phenotype of macrophages and analysis of SIRPα, FcγRI, and FcγRII surface expression.** (A) Primary human macrophages were stained for different surface markers as indicated and analyzed by flow cytometry. For each marker, specific staining (gray histogram) is compared with an isotype-matched control staining (black open histogram). (B–D) Cells were incubated for 10 or 30 min in wells coated with PLL (nonactivated) or hIgG, stained, and analyzed by flow cytometry. Expression of SIRPα (B), FcγRI (C), and FcγRII (D) in the live $CD14^+$ cells was assessed with APC-labeled specific antibodies (B and C) or with unlabeled primary antibody followed by isotype-specific secondary antibody conjugated with AF488 (D). For each receptor, representative histograms of specific staining (gray and black dashed open histogram) are compared with an isotype-matched control (black open histogram). Bars represent the geometric mean fluorescence intensity (gMFI) ± SD of three independent donors.

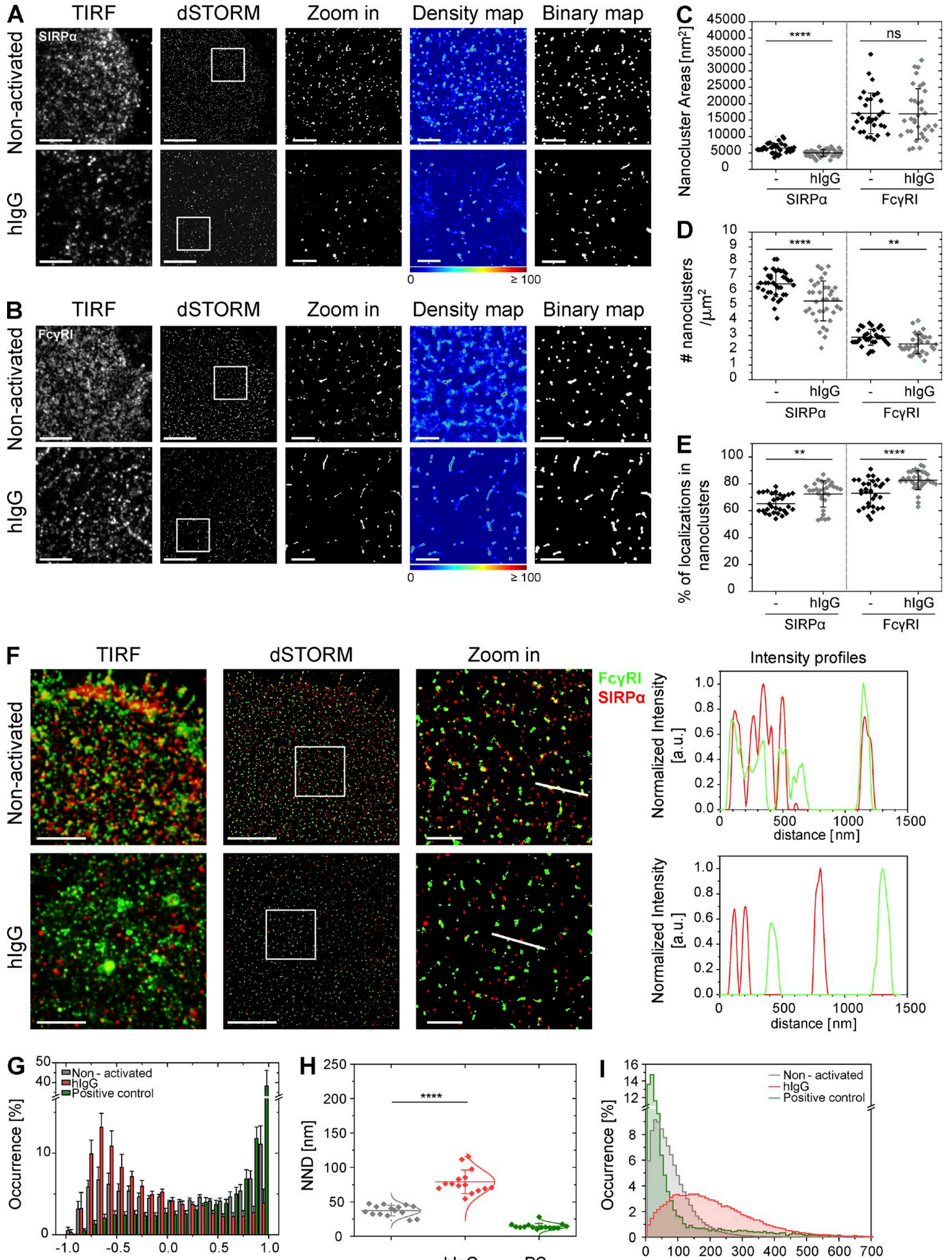

Figure S2. **Nanometer-scale organization of SIRPα and FcγRI at 30 min of stimulation.** (A and B) Representative TIRF and dSTORM images of SIRPα (A) and FcγRI (B) at the surface of primary human macrophages seeded onto PLL (nonactivated, top) or hIgG-coated slides (bottom) for 30 min and stained with fluorescently labeled specific antibodies. Bars, 5 µm. The regions delineated by the white squares are zoomed-in and shown with corresponding density maps and binary maps. Bars, 1 µm. (C–E) Nanocluster areas (C), density (D), and percentage of localizations in nanoclusters (E) for SIRPα and FcγRI under nonactivated (black) or hIgG-activated (gray) conditions were calculated as in Fig. 1. (F) Representative TIRF and dSTORM images showing FcγRI (green) and SIRPα (red) at the surface of primary human macrophages incubated for 30 min as in A and B and stained with anti–FcγRI-AF488 and anti–SIRPα-AF647 mAbs. Bars, 5 µm. Regions outlined by the white squares (middle column) are shown enlarged (right columns) with relative fluorescence intensity profiles along the white lines. Bars, 1 µm. (G) CBC histograms of the single-molecule distributions of the colocalization parameter for FcγRI and SIRPα in cells seeded onto PLL- (light gray) or hIgG-coated (light red) slides or in positive control (green). The positive control data in this figure is the same as in Fig. 2. Data are from a minimum of 15 cells from three independent donors. Bars represent mean ± SD. (H) NND analysis from data shown in G. Each symbol represents the median NND of all paired single-molecule localizations from one cell. Horizontal lines and error bars represent mean ± SD. Data are from a minimum of 15 cells from three independent donors. (I) Histogram distributions of the NND between the centroids of nanoclusters from one channel and the centroid of their nearest neighbor from the second channel (≥20,000 clusters from a minimum of 10 cells per condition) in PLL (light gray), hIgG (light red), or positive control (green) conditions. ns, not significant; **, $P < 0.01$; ****, $P < 0.0001$; two-tailed *t* test assuming unequal variance.

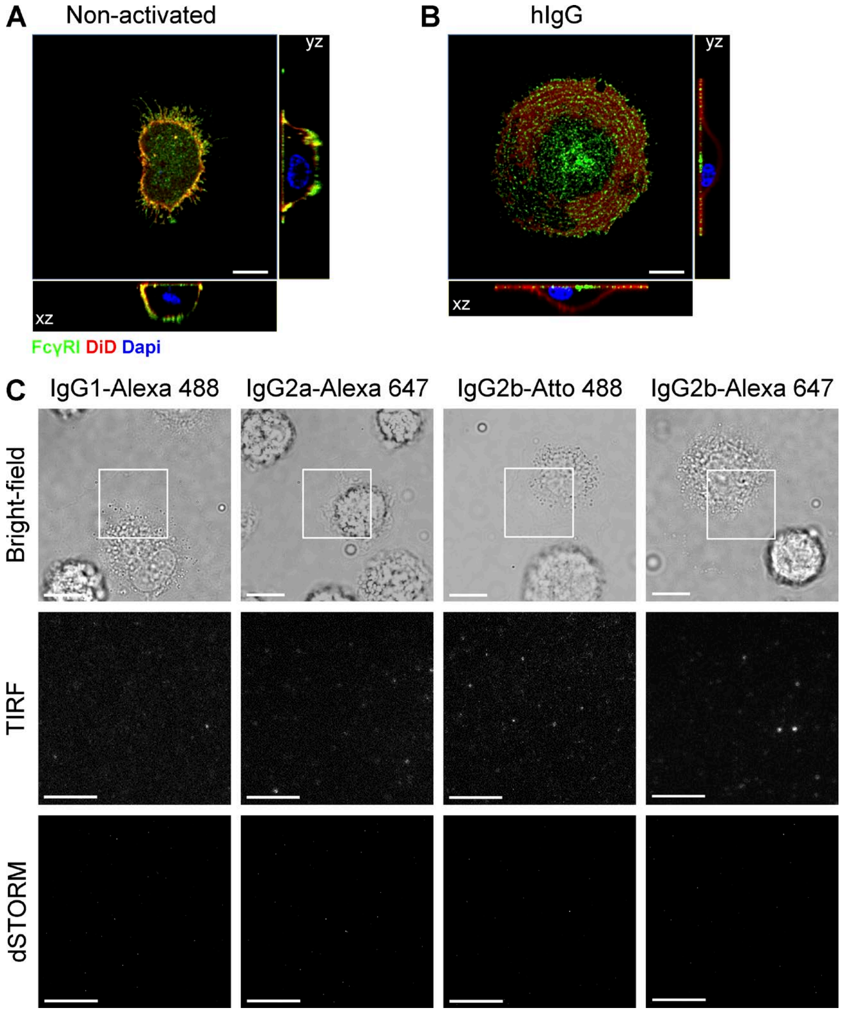

Figure S3. **FcγRI reorganization into concentric rings at the phagocytic synapse visualized by confocal microscopy.** (A and B) Representative confocal image of FcγRI at the phagocytic synapse and XZ and YZ orthogonal sections. Cells were allowed to spread onto PLL- (A) or hIgG-coated (B) slides for 10 min and then fixed and stained with an anti–FcγRI mAb conjugated with AF488 (green), the membrane dye DiD (red), and NucBlue Live cell stain (blue) to visualize the nucleus. Z-depth for nonactivated and hIgG conditions is 13 µm and 10 µm, respectively. Bars, 10 µm. See also Videos 3 and 4. (C) Isotype-matched control staining. Representative bright-field (bars, 10 µm) and TIRF and dSTORM images (bars, 5 µm) corresponding to the regions outlined by the white squares, showing primary human macrophages stained with isotype-matched control antibodies for all antibodies used throughout this study.

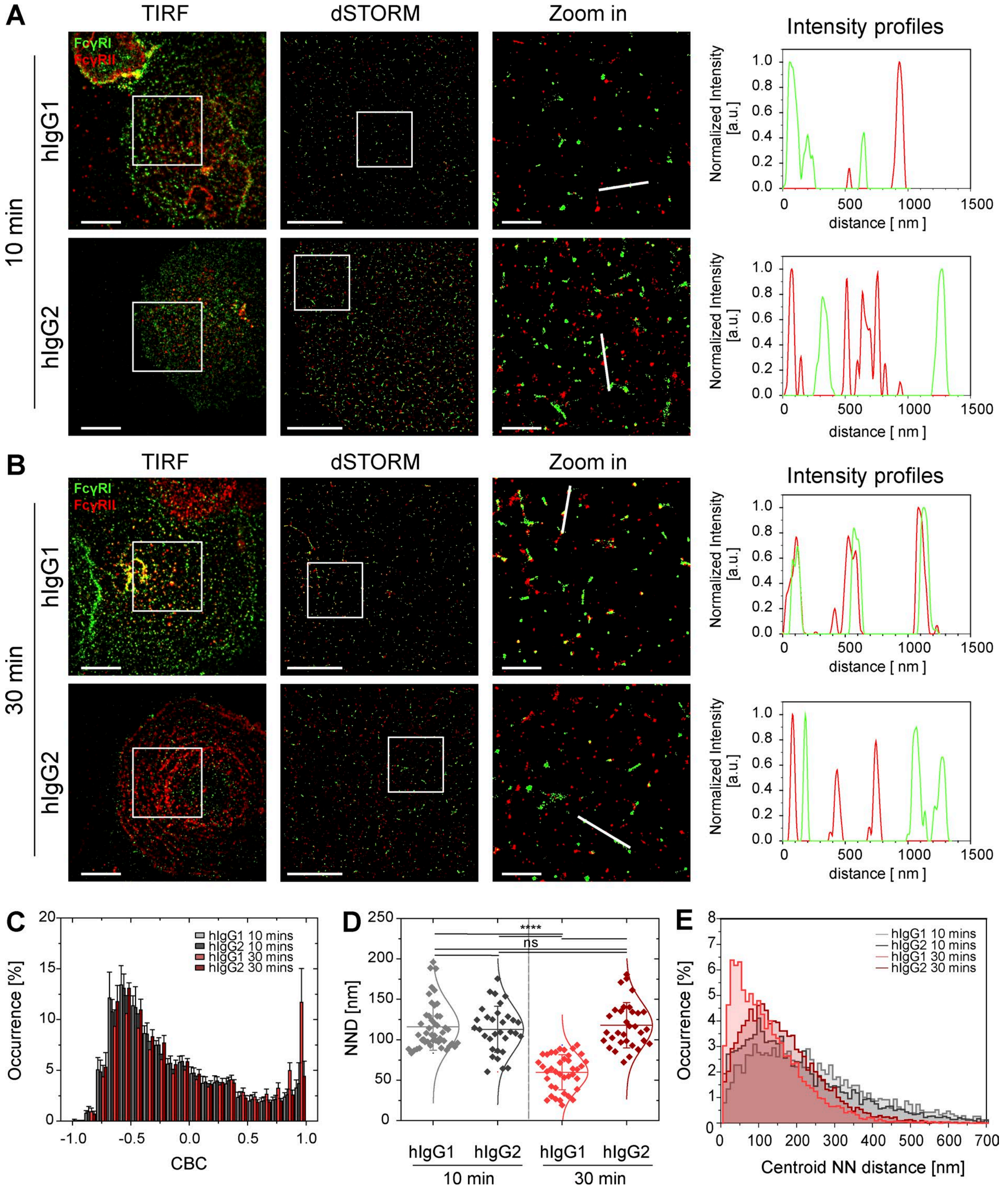

Figure S4. **Engagement of Fcγ receptors is required for their reorganization into concentric rings.** (A and B) Representative TIRF (bars,10 µm) and dSTORM images (bars, 5 µm) showing FcγRI (green) and FcγRII (red) at the surface of primary human macrophages, incubated for 10 min (A) or 30 min (B) on slides coated with hIgG1 or hIgG2, and stained with anti–FcγRI-AF488 and anti–FcγRII-AF647 mAbs. In each condition, regions outlined by the white squares (middle column) are shown enlarged (right column) with relative fluorescence intensity profiles along the white lines. Bars, 1 µm. (C) CBC histograms of the single-molecule distributions of the colocalization parameter for FcγRI and FcγRII in cells seeded onto hIgG1- or hIgG2-coated slides for 10 (light gray and dark gray, respectively) or 30 min (light red and dark red, respectively). Data are from a minimum of 20 cells from three independent donors. Bars represent mean ± SD. (D) NND analysis from data shown in C. Each symbol represents the median NND of all paired single-molecule localizations from one cell. Horizontal lines and error bars represent mean ± SD. ns, not significant; ****, $P < 0.0001$; one-way ANOVA with Tukey's post-hoc test. (E) Histogram distributions of the NND between the centroids of nanoclusters from one channel and the centroid of their nearest neighbor (NN) from the second channel (≥10,000 clusters from a minimum of 10 cells per condition).

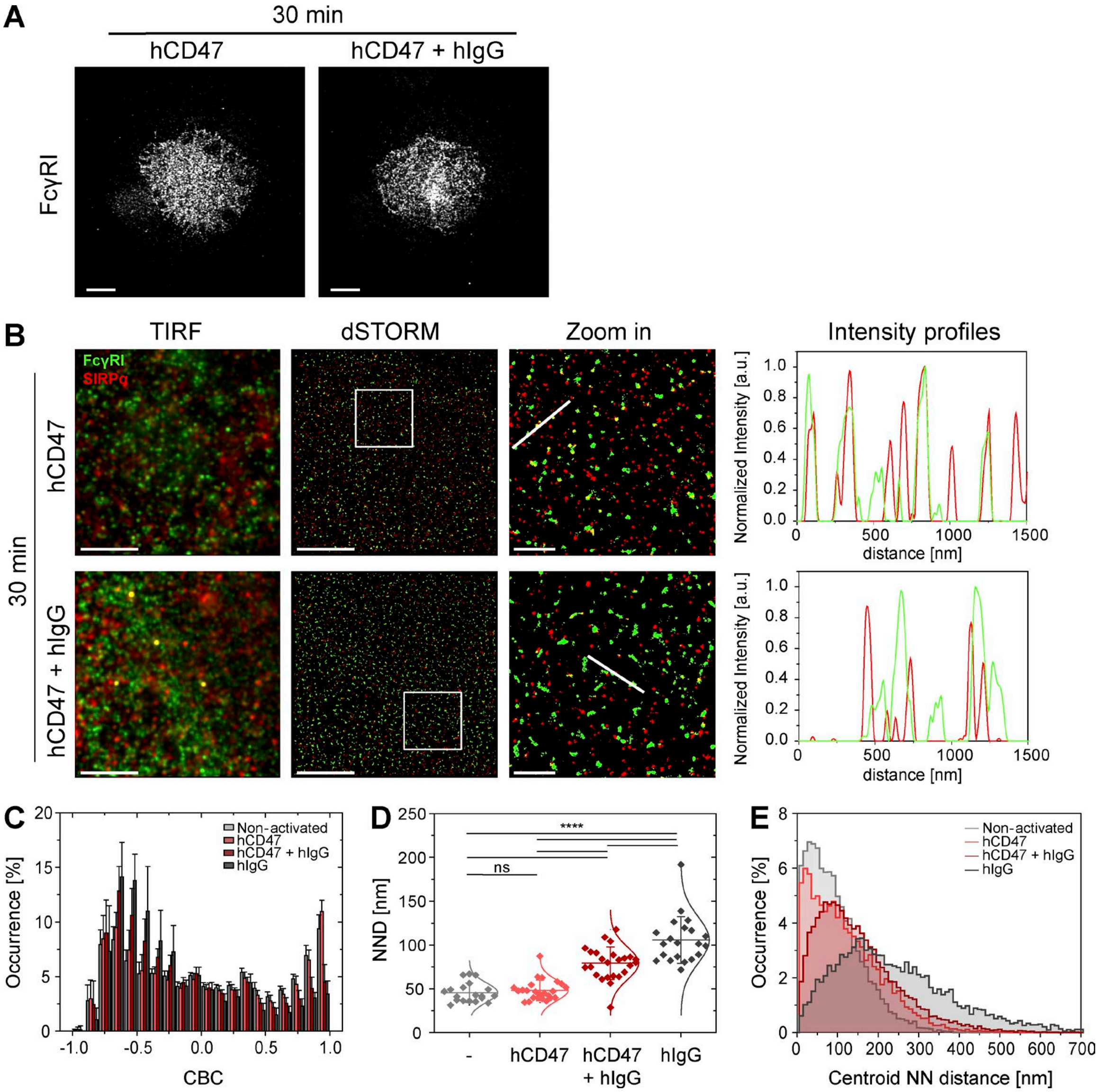

Figure S5. **SIRPα inhibition of FcγRI segregation and reorganization into concentric rings persists at 30 min of activation.** (A) Representative TIRF images of FcγRI at the surface of primary human macrophages incubated for 30 min on slides coated with hCD47 or hCD47 plus hIgG and stained with a fluorescently labeled specific antibody. Bars, 10 µm. (B) Representative TIRF and dSTORM images showing FcγRI (green) and SIRPα (red) at the surface of primary human macrophages incubated for 30 min on slides coated with hCD47 (top) or hCD47 plus hIgG (bottom) and stained with anti–FcγRI-AF488 and anti–SIRPα-AF647 mAbs. Bars, 5 µm. In each condition, regions outlined by the white squares (middle column) are shown enlarged (right column) with relative fluorescence intensity profiles along the white lines. Bars, 1 µm. (C) CBC histograms of the single-molecule distributions of the colocalization parameter for FcγRI and SIRPα in cells seeded onto slides coated with PLL (light gray), hCD47 (light red), hCD47 plus hIgG (dark red), or hIgG (dark gray) for 30 min. Data are from a minimum of 30 cells from three independent donors. Bars represent mean ± SD. (D) NND analysis from data shown in C. Each symbol represents the median NND of all paired single-molecule localizations from one cell. Horizontal lines and error bars represent mean ± SD. ns, not significant; ****, $P < 0.0001$); one-way ANOVA with Tukey's post-hoc test. (E) Histogram distributions of the NND between the centroids of nanoclusters from one channel and the centroid of their nearest neighbor (NN) from the second channel (≥10,000 clusters from a minimum of 10 cells per condition).

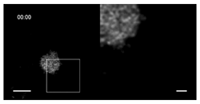

Video 1. **Live TIRF imaging of the distribution of FcγRI at the cell surface during the spreading of live primary human macrophages under nonactivating conditions.** Frame rate, 10 frames/s, sped up 20× from real time. Bars: 10 µm; (inset) 2 µm.

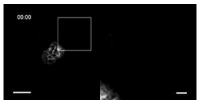

Video 2. **Live TIRF imaging of the formation of FcγRI concentric rings at the cell surface during the spreading of live primary human macrophages under activating conditions.** Frame rate, 5 frames/s, sped up 20× from real time. Bars: 10 µm; (inset) 2 µm.

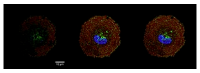

Video 3. **The video shows a representative confocal Z-stack and 3D projection of the distribution of FcγRI at the surface of primary human macrophages under activating conditions.** FcγRI, green; cell membrane, red; and nucleus, blue. Bar, 10 µm.

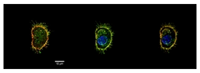

Video 4. **The video shows a representative confocal Z-stack and 3D projection of the distribution of FcγRI at the surface of primary human macrophages under nonactivating conditions.** FcγRI, green; cell membrane, red; and nucleus, blue. Bar, 10 µm.
